# From System Modeling to System Analysis: The Impact of Resolution Level and Resolution Distribution in the Computer-Aided Investigation of Biomolecules

**DOI:** 10.3389/fmolb.2021.676976

**Published:** 2021-06-07

**Authors:** Marco Giulini, Marta Rigoli, Giovanni Mattiotti, Roberto Menichetti, Thomas Tarenzi, Raffaele Fiorentini, Raffaello Potestio

**Affiliations:** ^1^ Physics Department, University of Trento, Trento, Italy; ^2^ INFN-TIFPA, Trento Institute for Fundamental Physics and Applications, Trento, Italy

**Keywords:** modeling, coarse-graining, molecular dynamics, proteins, biophysics

## Abstract

The ever increasing computer power, together with the improved accuracy of atomistic force fields, enables researchers to investigate biological systems at the molecular level with remarkable detail. However, the relevant length and time scales of many processes of interest are still hardly within reach even for state-of-the-art hardware, thus leaving important questions often unanswered. The computer-aided investigation of many biological physics problems thus largely benefits from the usage of coarse-grained models, that is, simplified representations of a molecule at a level of resolution that is lower than atomistic. A plethora of coarse-grained models have been developed, which differ most notably in their granularity; this latter aspect determines one of the crucial open issues in the field, i.e. the identification of an optimal degree of coarsening, which enables the greatest simplification at the expenses of the smallest information loss. In this review, we present the problem of coarse-grained modeling in biophysics from the viewpoint of system representation and information content. In particular, we discuss two distinct yet complementary aspects of protein modeling: on the one hand, the relationship between the resolution of a model and its capacity of accurately reproducing the properties of interest; on the other hand, the possibility of employing a lower resolution description of a detailed model to extract simple, useful, and intelligible information from the latter.

## 1 Introduction

Among the many revolutions that have spangled the 20th Century, the advent and diffusion of the computer is certainly one of the most momentous. Computing machines have impacted human life and society in practically all compartments, such as communication, work, information, education, health, and entertainment. The scientific environment is certainly one of the main leaders of this revolution, but it has been largely affected by it as well: in fact, computers have not only changed the way we do science, they also created new ways of doing science that were simply unthinkable before. Besides the “trivial” usage of computers in speeding up regular calculations (that is, to carry out the job of Los Alamos’ *human computers* ^1^ in a faster and more human-friendly manner), a novel technique arose that rapidly became pervasive of practically all scientific fields, as well as a field *per se*: computer simulations.

Among the synonyms of *simulation* we can find words such as *copy*, *facsimile*, *imitation*, *counterfeit*, and *fake*. Computer simulations are indeed all these things: while aiming at reproducing, as faithfully as possible, the real object of study, its properties, and its dynamics, they necessarily are but *the shadow of a dream*—the fictitious dance of a projection of the object. And yet, precisely in this intangible nature lies their power.

Simulations constitute a bridge between the experimental investigation of a system and its abstract, theoretical study. While the former relies on direct observation, probing, and quantitative measurement, the latter describes the system or phenomenon of interest in terms of quantities and relations among them, and carries out the investigation making use of mathematical manipulations. The computational approach takes from both: it presupposes a representation of the system in terms of rather idealized fundamental constituents, whose nature is closer to abstract Platonic entities rather than physical, “Aristotelian” ones. Such representation enables the investigation down to a level of detail that is practically and even fundamentally inaccessible to experiments; however, its usability in the study and comprehension of Nature presupposes that a one-to-one relation can be established between the constitutive elements of a real system and those of its *model*. The validity of the latter depends on the capacity of the modeler of identifying the essential features of a system and endowing the model with them; a model is just as good as the pieces of which it is made.

The field of application of this *computational microscope* (Lee et al., 2009) spans several orders of magnitude in space and time. Depending on the specific property or phenomenon of interest, various models can be employed that describe reality (or rather a part of it) in a relatively small length and time scale interval; no single model can be employed to study whatever system, for two reasons: our limited computational capacity, and the intrinsic limitations of the model.

At present, the most successful description we possess of the constituents of matter and their interactions is provided by the Standard Model of particle physics: even though the latter is an incomplete and effective^2^ theory, it still provides the most powerful and predictive (Hanneke et al., 2011; Aoyama et al., 2015) framework for the investigation of physical reality; that is to say, this theory constitutes the sharpest conceptual device we currently have at hand to rationalize observed phenomena and predict new ones. Nonetheless, a straightforward and brute-force application of such model to the study of systems larger than a small atomic nucleus is practically unfeasible: in fact, the associated computational cost makes it impossible to simulate, in terms of relativistic quantum fields, even the smallest molecule for a physically interesting time scale. Hence the first of the two aforementioned limitations.

All models beyond the most fundamental one (if any) are affected by both shortcomings. Certainly there will be systems too large or processes too slow to be studied by means of any derived representation; additionally, all these non-fundamental, effective representations will have a range of validity beyond which the model does not make sense. Non relativistic quantum mechanics works well for slow, low-energy particles, but the *resolution* of the processes it can reproduce is limited from below; additionally, it is too complex to study systems composed by more than a few atoms. Fortunately, within appropriate ranges of time, size, and energy, further effective theories can be constructed, that allow one to incorporate quantum mechanical properties in classical potentials: this process, epitomized by the Born-Oppenheimer approximation, fills the gap between quantum and classical mechanics, and between the small world and the not-so-small (e.g., molecular) world.

In general, then, the larger the scale of the system, and the longer the time scales of the processes of interest, the harder it is to perform simulations at a given level of resolution. This limitation originates from the increasing duration of the simulation and, in turn, the necessity to employ larger and larger memory and computing power. However, even when sufficient computational resources are at hand, another issue lies before us, which is the capability to make sense of the simulation. A detailed description of a large macromolecule, e.g., one in which each atom is described as a point-like particle, is certainly sufficient to reproduce several properties that would not involve quantum mechanical features explicitly, but it might as well be *excessively detailed* for the purpose. A simplified representation of the system and its interactions might be sufficient to reproduce the process of interest.

A further reduction of resolution is thus possible, in which the system is not described in terms of atoms, but rather of effective interaction sites each of which is representative of a group of several atoms. A model whose resolution is lower than atomistic is commonly referred to as a *coarse-grained* (CG) model. CG models range all possible resolutions from a few atoms per site up to the continuum, and a plethora of strategies have been developed to parametrize them so as to reproduce one or more properties of the system of interest. In fact, exactly as any effective theory can be trusted in a limited range of length and times scales only, so it is for any particular CG model.

**FIGURE 1:**
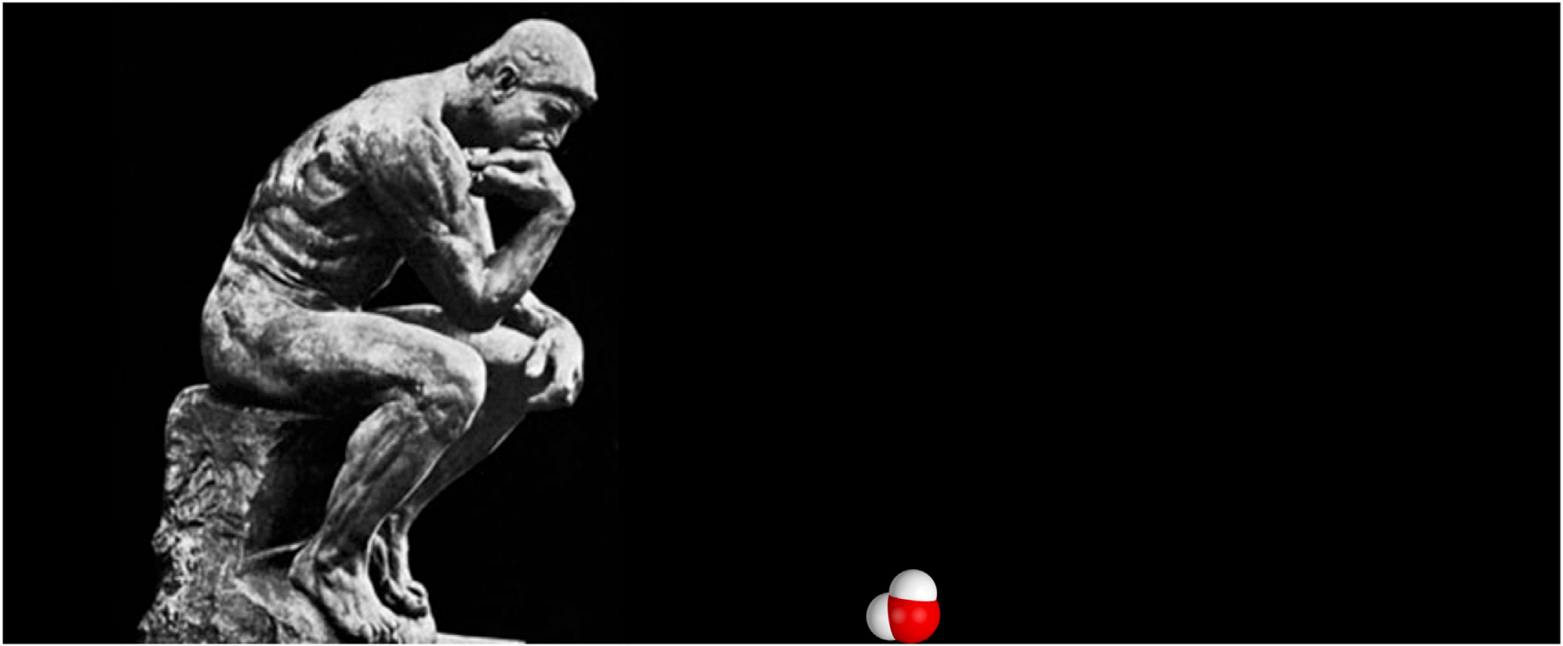
In the construction of a model we are confronted with several questions, whose apparent philosophical quality entails a rather practical nature. In particular, we ask ourselves: Can one always coarse-grain? Is there an optimal level of resolution? Is a single level of resolution meaningful? How to identify the optimal resolution level or distribution? And how can we implement it in practice? (In the picture: “The thinker”, A. Rodin, 1904).

This apparently trivial observation opens up a crucial issue, whose practical and philosophical implications have just started to be studied (see Figure 1), namely the identification of the level of model detail that is the most appropriate for the study of a given phenomenon. In fact, the construction of a model is implicitly dictated by its purpose, and its usage implicitly complies (or should comply) with the range of validity in which the model is effective. Insofar, the decision of the model resolution has largely been based on intuition, and quantitative investigation of the appropriate level of detail is really just in its infancy.

A second, even more subtle issue is the definition of the appropriate resolution *distribution*, that is, whether each system part should be represented with the same level of detail, or rather a modulation of the latter can be implemented so as to attribute higher resolution (and more computational resources) to a given region, while reducing the accuracy elsewhere. This task actually requires solving two problems: first, one has to determine what level of resolution can be employed, and where; second, one has to devise a model that guarantees the appropriate degree of accuracy to each region of the system, such that the various regions at different resolution can interact with one another seamlessly.

Besides the questions related to the construction of computational models of physical systems a further one lies, that tackles the issue of system representation from a different perspective, namely that of employing modeling strategies for the *analysis* of the system. Computer simulations of large systems are becoming increasingly more feasible, which bears with it two major consequences: on the one hand, the steady growth in the amount of data to make sense of, even for a single run; on the other hand, the increase in the complexity of the systems and processes that can be tackled, which naturally requires a richer and often system-specific toolbox of analysis instruments. These are essential to safely navigate the sea of data produced, and land to the shores of the system’s *understanding*; the latter, however, can only be achieved through a process of *reduction and synthesis*, by which the vast amount of numbers crunched and spat out by the computer are distilled into a few, intelligible and interpretable parameters, their time evolution, and their relations.

This is indeed what is being done since the dawn of thermodynamics, as systems composed by bazillions of particles are eventually described and studied in terms of a handful of quantities (temperature, pressure, volume, chemical potential, compressibility, specific heat...). The necessity behind this procedure is the human incapacity of making sense of $∼10^{23}$ degrees of freedom; the reason behind the success of such a drastic program, which brings down that number of coordinates to less than ten, is the fact that indeed a full, *meaningful* characterization of the system is intrinsically achievable in terms of those few variables and no more than that.

In the case of a system as simple as a gas or a liquid, the identification of those parameters that are relevant and sufficient for a complete description and understanding of the system is straightforward and largely intuitive. When the object of study is a macromolecule, however, things might be more subtle: one can wonder if it is possible to devise an algorithmic procedure aimed at the identification of those variables in terms of which a simplified representation of the system can be achieved, which maximizes the insight about it while at the same time retaining the lowest number of descriptors. Questions such as this hold the promise of discriminating, in an unsupervised manner, the signal from the noise in the outcome of a computer simulation.

The scope of this review is to present and discuss in some detail the questions raised insofar. The extension and richness of the field of modeling and coarse-graining forces us to renounce at any expectation of exhaustiveness: we however hope to provide the readers with a sufficiently broad and organized overview to grasp and appreciate the variety and diversity of models and methods that have been developed in the context of computer simulations of macromolecules. Our focus will lie on applications to proteins. This choice has two reasons: first, any attempt at including more than this class of systems might have easily doubled the length of the manuscript, as the field of biological and artificial soft matter modeling is just as broad as the list of systems itself; second, all the issues we discuss find in proteins a most evident, remarkable, and interesting playground. Many problems that we pose make little to no sense in other contexts: for example, it is relatively uninspiring (even though not devoid of insight (Harmandaris et al., 2006; Ohkuma and Kremer, 2017)) to wonder about a modulation of the model resolution in a long homopolymer, or to question whether an intrinsically optimal level of detail exists in the representation of a lipid bilayer. On the contrary, these questions can have as many different answers as the proteins they are applied to, due to the diversity of size, structure, function, and properties that these molecules exhibit.

The remainder of the paper is structured as follows. In Section 2 we introduce some fundamental concepts upon which the procedures of modeling and coarse-graining are constructed. Albeit non-standard and universally accepted terms are introduced, these are sufficiently intuitive and serve the purpose of removing some of the potential ambiguities that such a broad and rich field entails. In Section 3 we discuss the most fundamental models one commonly finds in the context of soft and biological matter simulation, namely atomistic models. We enter the field of coarse-graining in Section 4, where we recapitulate the main general ideas, and illustrate examples of the models and methods that have been developed. Specifically, in Section 5 we focus on those models where the degree of detail is uniform through out the system, while in Section 6 we consider those strategies that make use of two or more resolution levels in the same model. In Section 7 we shift from the idea of modeling to that of *filtering*, that is, reading a simulation with a lower level of detail so as to discriminate those structural characteristics that entail the largest amount of information about the system properties. Finally, in Section 8 we summarize with a few concluding thoughts.

## 2 Representation, Model and Filter

In order to carry out some kind of computer-aided quantitative investigation of a macromolecular system, e.g., a molecular dynamics simulation, it is necessary to provide a *representation* of the system. By this term we refer to a collection of mathematical entities conceptually associated to a corresponding physical entity: for example, the physical entity “atomic nucleus” is associated to a point in three dimensions whose position in space is determined through its coordinates in a (usually three-dimensional) Cartesian space. This point is the mathematical entity associated to the physical atomic nucleus, and a collection of such points, one for each atom of the molecule and its environment, constitutes the representation of the system we feed the computer with.

As such, the representation is a static object. This does not mean that it cannot be informative *per se*: indeed, knowing the position of the molecule’s atoms allows us to establish geometrical relationships among them, from which, in turn, we can infer properties that connect shape and function. A particularly intuitive example is provided by the crystallographic or NMR techniques that turn real molecules in a set of atomic coordinates. However, in order to make a step forward from structure to function it is necessary to expand the set of properties the representation is endowed with and, most importantly, to confer to it the capacity of actively producing dynamic information. To this end, we have to “dress” the representation with *interactions*, thus enabling it to evolve in time or to sample its accessible phase space. When properties and interactions are specified for a given representation of the system, we dub it a *model*. Models are thus mathematical idealisations of a system that, by means of an appropriate processing of their properties and interaction, can produce nontrivial information (e.g., time series, correlation and response functions, conformational sampling...).

**FIGURE 2:**
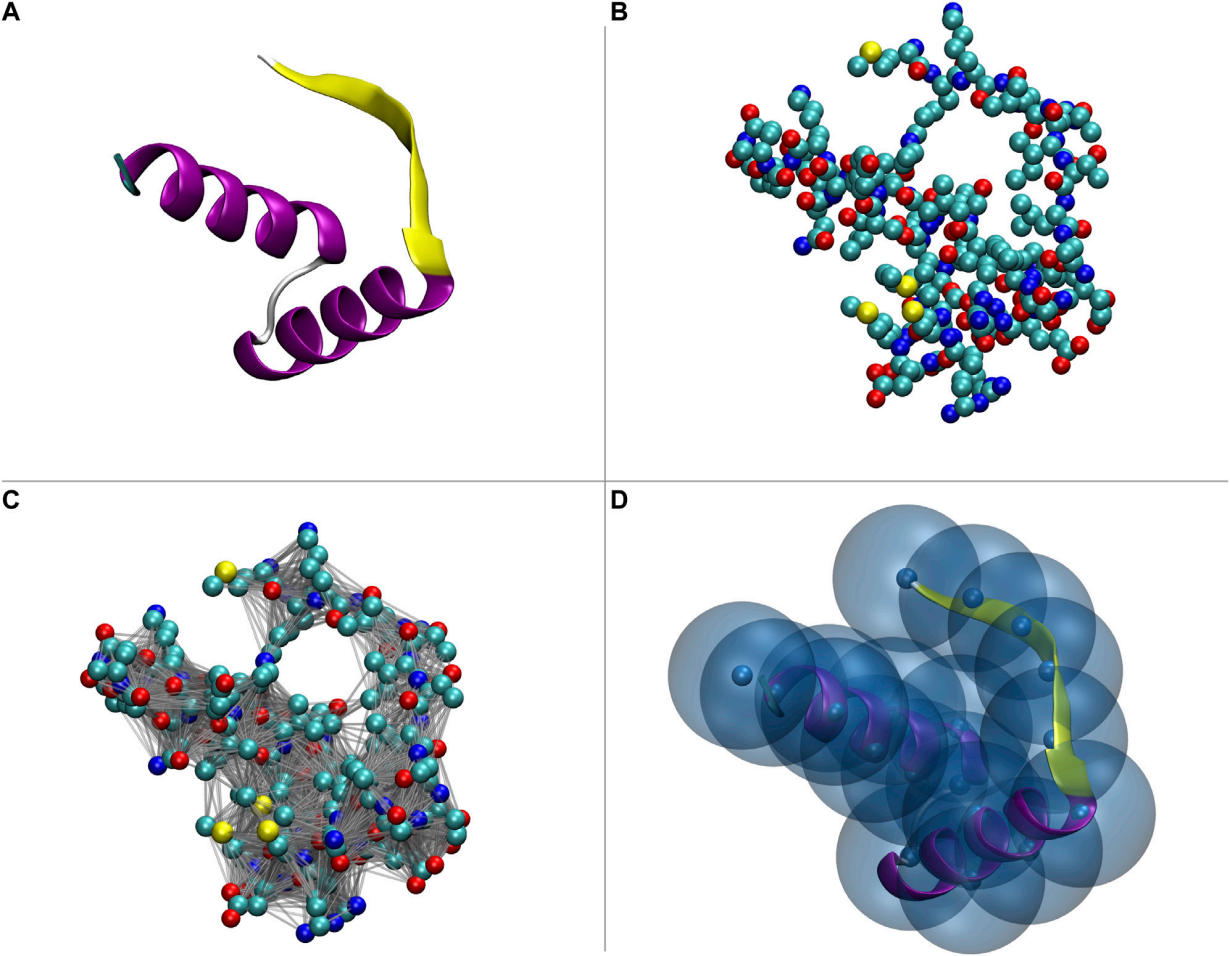
Pictorial illustration of modeling and filtering. A real system, such as a protein (panel **(A)**, PDB code 2CPG) can be described in terms of the position of its atoms (panel **(B)**). If we assume it to be the most detailed representation, this constitutes the *first level representation* (FLR). By “dressing” it with interactions we obtain a *model* (panel **(C)**) that can be used to perform conformational sampling, e.g., through a molecular dynamics simulation. The resulting conformations can be inspected with the same level of detail of the FLR; alternatively, only a subset of the model’s degrees of freedom can be taken into account: in this latter case we have a lower-resolution, *second level representation* (SLR), that is obtained through the application of a *filter* to the FLR (panel **(D)**).

Once a representation or a model are given, though, one can apply to them the same procedure that leads from the real-life system to the idealized representation. More specifically, given a representation that we think of as the more fundamental one, thus dubbed *first level representation* (FLR), it is possible to establish a quantitative relationship from its mathematical entities to those of a *second level representation* (SLR), typically given in smaller number than those of the first one. As an example, all the atoms constituting a protein in the first level representation can be associated to one single point for each amino acid, so that the second level representation constitutes a *simplified* description of the first. If this procedure is applied to all configurations obtained in a molecular dynamics simulation, the result is a trajectory generated with the model defined at the first level, but described in terms of the second level representation. Hence, the SLR cannot produce nontrivial information by itself, but it can return a *subset* of the information produced by the underlying model; because of this property we refer to such SLR overlaid on a model as a *filter* ^3^. These ideas are illustrated in Figure 2.

For a given system, one can provide representations at different levels of resolution: restricting our considerations to particle-based representations of proteins, we can let each of these particles represent an atom, part of an amino acids, an entire amino acid, a group of amino acids, an entire protein and so on. When the size of the system is such that a particle-based description of it does not make sense any more, continuum or quasi-continuum representations come into play, such as finite elements representations, where the surface of a protein is described in terms of a triangular tessellation, or descriptions involving density fields. Each of these representations is informative in its own right, in that it can highlight different structural features of great importance—atom proximity, binding pocket geometry, solvent-accessible surface area, overall shape, and so on. As already highlighted, however, the amount of information they can deliver is limited to what can be extracted from the structure alone; to gain further insight, conformational sampling, time correlation and energetics are required, which can only be achieved through simulation and, in turn, rely on a set of interactions. Each of the aforementioned representations thus constitutes the basis for a wide range of models, differing by complexity, accuracy, computational requirements, and so on. These models are employed to investigate the behavior and properties of systems at various level of detail, ranging from all-atom descriptions, where each atom is explicitly accounted for, to very coarse and qualitative pictures where an entire protein is treated as a featureless sphere. Evidently, the choice of a model over another depends on the problem one is interested in: the resolution of the model determines the lowest-level, most fundamental causes it can produce, and with it the processes and properties it can generate.

Filters, on the other hand, have not been developed so far with the same intensity as models, in spite of the fact that their usage is ubiquitous. We cannot think of making sense of an all-atom molecular dynamics simulation by examining all 3N coordinates in each frame at a time: a process of *synthesis* ^4^ is necessary in order to extract, from such a large amount of data, the relevant *and intelligible* bit that we can make use of. This process is often carried out quantitatively, e.g., by defining a specific reaction coordinate that allows one to discriminate between two distinct conformers of a molecule. In such a case, one has to know in advance what to look at in order to construct this coordinate; rather frequently, however, a qualitative approach is the first and possibly unique one, and it takes the form of a *visual inspection* of the MD trajectory. Albeit very sophisticated, as it passes through immensely complex neural networks (our brains), the information is eventually reduced to simple notions such as “open” and “closed”.

More quantitative examples of filters are available, such as simplified representations of a protein in terms of quasi-rigid domains, where a group of amino acids is treated as a unique block whose internal dynamics is neglected. To determine the structure of these domains (i.e., which amino acids belong to which domain) on the basis of their dynamical properties it is necessary to make use of a model defined at a higher resolution with respect to the blocks themselves; however, once their identity is fixed, the trajectory can be studied *filtering out* the movement internal to the blocks and focusing on the relative displacements among them. This procedure of feature extraction enables one to derive, from a large amount of data inherent in the output of a model defined at the fist level of representation, a smaller and more manageable amount of information defined at the second level of representation.

In the following, we will provide an overview of the most common models employed in the computer-aided investigation of proteins, and discuss what impact the level and distribution of the detail of the underlying representation has on the capacity of the model to generate information; subsequently, we will discuss how filters can be employed to rationalize the impressive amount of data produced in a single MD run and separate the signal from the noise.

## 3 All-Atom Modeling

The models of reference in the computational study of molecular systems are the so-called *all-atom* models. They are defined at an atomic scale resolution, meaning that each atomic nucleus is represented as a material point-like particle.

In all-atom models, each particle interacts with the surrounding ones through classical potentials. This is justified by the Born-Oppenheimer approximation (Born and Oppenheimer, 1927), which allows one to eliminate the electron degrees of freedom by taking the quantum-mechanical expectation value over the electronic wave function, under the assumption of an effective decoupling of nuclear dynamics and electronic ground state. As a result, the interaction energy, which is quantum in nature, can be approximated by a classical potential energy surface that depends only on the position of the atomic nuclei, ignoring the evolution of the electronic distributions. Moreover, interactions in all-atom models are based on the assumption that the contribution of each atom to the Born-Oppenheimer potential energy can be approximated by a sum of few-body terms, each shaped into a simple, empirical and semi-empirical functional form. These energy terms, which collectively take the name of *force field*, can generally be divided in two types: those describing bonded interactions, and those describing interactions between particles close in space but not connected by any chemical bonds (non-bonded interactions). The former are associated to the presence and distortion of chemical bonds, and are modeled as a sum of contributions with a dependence on bond lengths, bond angles, and dihedral angles; non-bonded interactions, instead, are described in terms of Van der Waals, electrostatic, and hydrogen bond potentials.

Force field parameters are obtained from experimental data and quantum-level calculations performed on specific sets of systems. Bond lengths and corresponding stiffness values, as well as angle parameters, are commonly determined from crystallographic or spectroscopic data; Van der Waals terms from small molecules liquid density, heat of evaporation, or solvation free energies; partial atomic charges from quantum-mechanical calculations (González, 2011). As no unique parametrization strategy exists, a plethora of atomistic force fields have been developed through the years, all having a strikingly similar functional form but different coefficients. In the case of proteins, examples of common atomistic force fields are Amber (Maier et al., 2015) and CHARMM (Huang and MacKerell, 2013); recently, improved versions of these force fields for both folded and intrinsically disordered proteins have also been developed (Huang et al., 2017; Robustelli et al., 2018), in addition to force field types designed for amyloid assembly (Nguyen et al., 2021). In any of these force fields, each amino acid type in a defined protonation state is described through the same set of parameters, irrespective of its position along the protein sequence; exceptions are the N- and C-termini, which usually require *ad hoc* parameterisations according to the capping groups. Moreover, particles within each residue are generally not distinguished on the basis of the sole chemical element, but according to the *atom type*. This distinction is much stronger than the one based on the atomic number, since atom types differ also in their hybridization state and the local electronic environment of the atoms they are covalently bonded to. The definition of atom types in a force field is of fundamental importance, since it determines the specificity of the interactions.

Even within the limits of validity imposed by the aforementioned approximations, these models are of tremendous importance to perform an *in silico* exploration of a macromolecule’s energy landscape, with the aim of bridging the gap between structure, dynamics, and function. In this regard, the conformational sampling method of choice in the biophysics community is molecular dynamics (MD), through which successive configurations of the system are generated by numerically integrating Newton’s equation of motion, thus allowing the calculation of both equilibrium and time-dependent properties.^5^

Atomistic MD has brought a significant progress in a wide range of biological applications in the last decades, due to the advancement of novel algorithms and high-performance computing. The gap between timescales resolved in simulations and in experiments has been significantly reduced due to the concurrent advances in the corresponding techniques; particularly significant is the recent diffusion of graphic processing units (GPUs) for MD calculations (Stone et al., 2010; Lindert et al., 2013; Sweet et al., 2013), and the consequent GPU implementation of popular molecular modeling software packages (Lee et al., 2018; Kutzner et al., 2019; Phillips et al., 2020). Groundbreaking was the development in the last decades of the supercomputer Anton (Shaw et al., 2008; Shaw et al., 2009; Shaw et al., 2014), specifically designed for running atomistic MD simulations with extremely high efficiency (Pan et al., 2016; Masureel et al., 2018; Pan et al., 2019).

Making use of standard resources, computational scientists can nowadays access micro-to millisecond timescales with atomic detail, for systems comprising several hundreds of thousands of particles. This is sufficient to characterize many critical biological processes, such as ligand-binding events and the folding of small proteins (Kubelka et al., 2004; Freddolino et al., 2008). However, several phenomena are still inaccessible with all-atom MD; this is the case of large-scale structural rearrangements, whose characteristic time scale typically impairs an exhaustive exploration of the accessible conformational space. To alleviate this limitation, diverse enhanced sampling techniques have been developed, including metadynamics and replica exchange MD, which boost the conformational sampling by “helping” the system overcome high free energy barriers; excellent reviews on these topics can be found in Bernardi et al. (2015) and Yang et al. (2019).

The advances in the field of atomistic simulation are paralleled by the increase in the amount of information they generate. MD trajectories, which consist of the set of three Cartesian coordinates per atom per simulation time step, can easily result in an enormous quantity of raw data: appropriate tools are required to separate the most meaningful information buried in the high dimensional space of the simulation output from the rest, thus addressing specific questions about the phenomenon under investigation. Indeed, this challenge led to the development and application of several dimensionality reduction algorithms (Sittel and Stock, 2018; Tribello and Gasparotto, 2019) to the analysis of all-atom MD trajectories. Still, no standard procedure or unique technique exists that can allow the blind and automated determination of the fundamental degrees of freedom of the system. Nowadays, the common approach consists in combining several analysis tools in a system-specific fashion, in order to reduce data complexity and facilitate the understanding. The specific analyses performed are strictly connected to the molecule simulated, to the technique used to generate the dynamics, and to the type of information one is interested in.

In order to investigate the behavior of a protein in terms of its structural stability, it is standard procedure to compute the root mean square deviation (RMSD) and the root mean square fluctuation (RMSF) on the positions of a subset of particles, typically the C_*α*_ atoms. The former quantity indicates the global evolution in time of the atomic position, and gives indications on the drift from a given conformation; the latter instead is usually time-averaged on a residue basis, and can help to identify the relative flexibility of protein segments. In addition, secondary structure content can be monitored by applying analysis algorithms such as STRIDE (STRuctural IDEntification) (Frishman and Argos, 1995) or DSSP (Define Secondary Structure of Proteins) (Kabsch and Sander, 1983), which assign a secondary structure conformation to each residue on the basis of the hydrogen bond pattern of its backbone. In the case of the STRIDE method, the secondary structure assignment includes torsion angle potential calculations, as well as statistical propensities extrapolated from experimentally determined structures. The information provided by DSSP and STRIDE is useful to follow the evolution of the secondary structures in time, and eventually to detect changes associated to partial unfolding or disorder-to-order transitions (Lin et al., 2019).

The combination of RMSD, RMSF, and secondary structure analysis can give details on specific regions of the protein that are more stable than others. As an example, in recent works (Spagnolli et al., 2019; Spagnolli et al., 2020), one of us investigated the stability of atomistic models of infectious prion proteins by combining the aforementioned analysis techniques in a synergistic approach. Due to difficulties in the wet lab procedure, experimentally solved structures of prion proteins are still unavailable; therefore, testing the stability of models via MD simulation represents an extremely important step toward the 3D structure elucidation.

If the protein under investigation undergoes large conformational changes, it can be appropriate to group, or cluster, the configurations explored during the simulation on the basis of their structural similarities. To this aim, various clustering methods have been developed in the past 30 years, each presenting different algorithmic characteristics and computational performances (Shao et al., 2007). The 2D RMSD matrix, which includes the deviations between any pair of trajectory frames, typically defines the distance between the conformations to cluster. Based on this measure, a variety of open source tools are available to perform the clustering; among these, it is worth mentioning the widely used MDAnalysis package (Michaud-Agrawal et al., 2011), which employs python libraries to perform the calculations.

Clustering can also be combined with principal component analysis (PCA), as in Wolf and Kirschner (2013) and Wolf and Kirschner (2013), where the two approaches are applied to the trajectory of a bacterial ribosomal domain. The advantages of applying PCA prior clustering analysis lie in the remarkable dimensionality reduction, which results in a simplification of the clustering operation, and in a better visualization of the clusters when plotted in the most represented PCA space.

While clustering allows one to easily identify the variety of conformations sampled during an MD simulation, it can hardly give information on the dynamics of transitions between them. Kinetically relevant states and their rates of interconversion can instead be estimated from Markov state models (Chodera and Noé, 2014). Starting from large sets of individual short MD trajectories, this approach has been used to tackle biological problems happening at relatively long time-scales, such as protein folding, protein-ligand binding, or large conformational changes.

Tools from information theory can also be employed to analyze atomistic trajectories. For instance, cross-correlation or mutual information (Lange and Grubmüller, 2006) can shed light on concerted movements between protein regions; while the former captures only linear correlations between residues, the latter can detect also the non-linear ones. Both cross-correlation and mutual information can be used to build a network representation of the protein, where each residue is defined as a node and the graph edges between neighboring elements are weighted according to the correlations extracted from an MD simulation. The resulting network can be analyzed through well-established techniques of graph theory, such as node centrality and edge betweenness (Böde et al., 2007). These analyses proved valuable in the study of allostery, as described in the work of (Bowerman and Wereszczynski, 2016). Here, the authors simulated at atomistic level the enzyme thrombin, and calculated correlations among residues in terms of cross-correlation, mutual information, and non-linear generalized correlation. The latter is then employed to construct a graph and obtain information about allosteric pathways and hotspots.

In spite of the advancements in simulation tools and analysis techniques, however, the simulation of large macromolecules and slow biophysical processes still remains out of reach for detailed, atomistic models. Additionally, such a high level of detail in the description of the system can even represent a limitation in the comprehension of the system of interest and its properties. To overcome these limitations simpler representations are employed, which offer an increased efficiency at the expenses of a lower degree of resolution. These *coarse-grained models* are the object of the following sections.

## 4 Coarse-Grained Modeling: General Framework

In 1975, Levitt and Warshel published a paper in which they employed an extremely crude representation of a protein in terms of few sites endowed with simple interactions to gain insight in the process of protein folding (Levitt and Warshel, 1975); while the specific results obtained have been later questioned (Hagler and Honig, 1978), this work represents a milestone as the first, pioneering attempt to investigate fundamental biophysical problems making use of minimalistic models of the system instead of extremely accurate ones. Since then, biomolecular CG modeling has steadily grown to become an essential tool in the computational investigation of biological matter: only considering proteins, a whole zoo of CG models and techniques has been developed, which aim at capturing the physicochemical behavior of a large variety of molecules over a wide range of characteristic length and time scales (Saunders and Voth, 2012; Kmiecik et al., 2016; Singh and Li, 2019; Nguyen et al., 2021). Given this extreme diversity, we deem it useful to briefly recapitulate the main concepts underlying the development or choice of a CG model.

**FIGURE 3:**
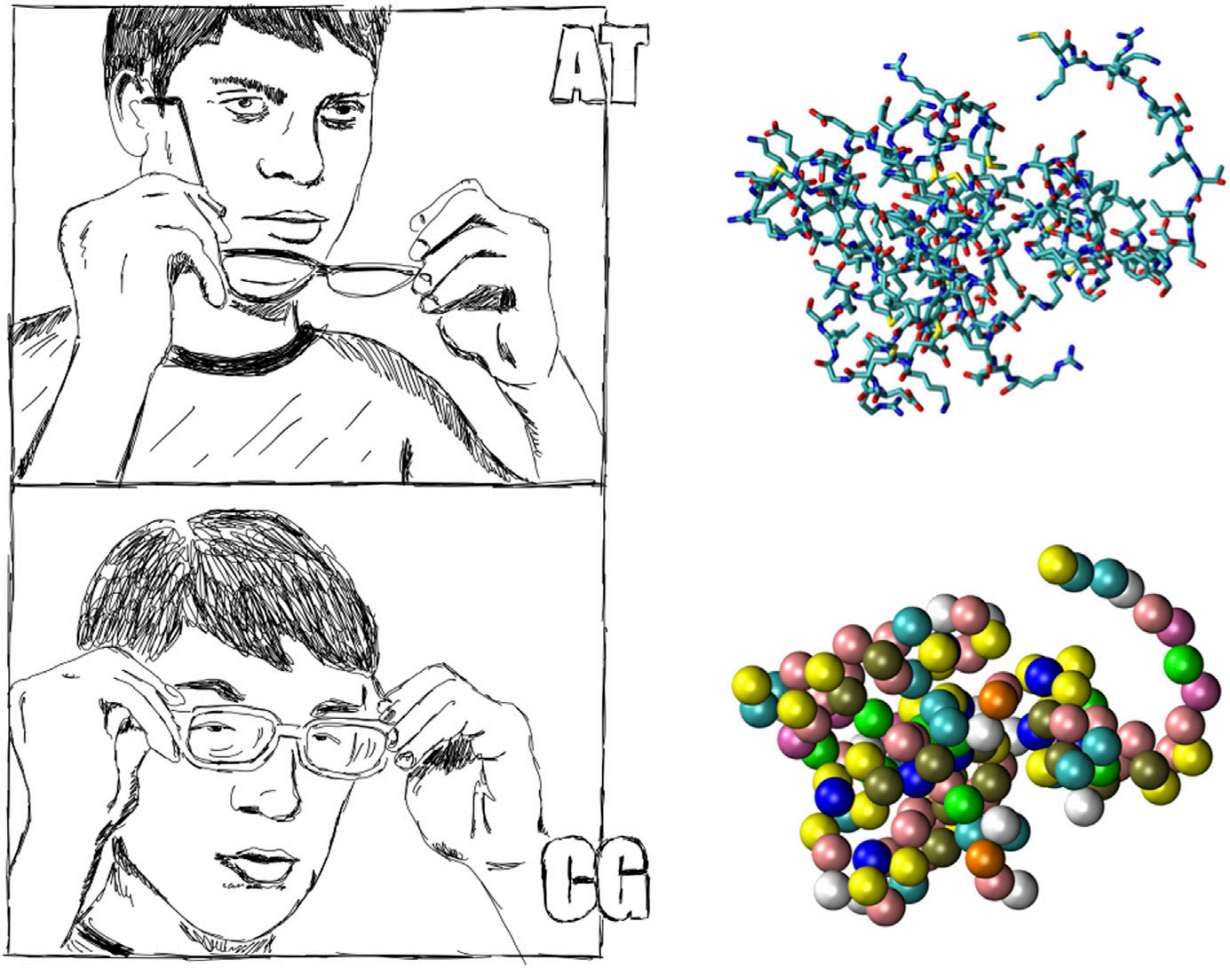
The process of filtering is akin to wearing a pair of spectacles while enjoying a perfect eyesight. The loss of detail is generally considered a defect, however it does have the advantage of simplicity and parsimony - if it is done properly. Artwork by R. Potestio.

As discussed in Section 2, the first ingredient required in the construction of a CG model for a biomolecular system is the selection of a SLR, obtained by superimposing a *mapping* to the fundamental representation—in our case, the atomistic one. The mapping constitutes the observational filter connecting the detailed description of the protein’s instantaneous configuration to its low resolution counterpart, meaning that, in the latter, only a limited amount of the original degrees of freedom is explicitly employed. One can think of this process as putting on a pair of “coarse-graining glasses” whose effect is that of blurring an already neat and defined image (see Figure 3).

Critically, inherent to the CG mapping is the definition of the elemental units composing the newly-introduced representation: in particle-based CG pictures (Noid, 2013; Kmiecik et al., 2016), such units are the effective interaction sites, or “beads”, obtained by lumping together subsets of the system’s constituent atoms. Depending on the chosen resolution level, each site can be representative of small to medium-sized chemical moieties (Monticelli et al., 2008; Bereau and Deserno, 2009; Darrè et al., 2015), single or groups of amino acids (Clementi et al., 2000; Atilgan et al., 2001; Micheletti et al., 2004; Zhang et al., 2017), up to entire molecular structures (Chu and Voth, 2006; Sept and MacKintosh, 2010; Dama et al., 2013). In all these cases, the mapping is formally expressed as the functional relation R=M(r) between the effective sites’ coordinates **R** and the atomistic ones **r** (Noid et al., 2008a; Rudzinski and Noid, 2011).^6^ In continuous or quasi-continuous CG representations (Oliver et al., 2013; Welch et al., 2020), the elemental units can instead be identified with the finite volume elements employed to decompose the protein’s macroscopic structure, and the mapping can be considered as the specific discretization mesh prescription employed in numerical calculations.

The selection of the level of resolution employed to describe a system is *per se* an already highly nontrivial problem, as this process naturally introduces a lower bound in the length scales the CG representation is in principle able to resolve. Indeed, for a CG observer it will be impossible to capture fluctuations of the system taking place below the size of the average radius of a bead—or the distance between two beads—in particle-based pictures, or smaller than the size of the discretization mesh in continuous ones. In turn, this implies that a CG representation characterized by a specific minimum length scale, when employed to *inspect* the system, can only enable the investigation of emergent properties or phenomena occurring *at or above* such scale: it is technically impossible for the CG filter to grasp the rotation of a specific protein side chain around its main axis, if it is depicted as a point-like particle. This limitation has to be explicitly accounted for when designing the low-resolution representation of a system.

Subsequently, for the simplified representation to acquire predictive power—that is, for the CG mapping to become a *model*—interactions among its effective degrees of freedom must be introduced. In the case of continuous CG representations, this amounts at providing, as input parameters, the appropriate material properties of the protein, e.g., shear viscosity and shear/bulk moduli, which determine the overall stress tensor of the system and consequently its hydrodynamic behavior (Oliver et al., 2013). Particle-based CG models, on the other hand, require the definition of the interaction potential—more precisely, a free energy—acting among the point-like effective sites that constitute the molecular structure (Rudzinski and Noid, 2011; Noid, 2013). As during the last decades substantial effort has been devoted to the development and application of particle-based CG protein models, we here showcase the main approaches behind the parameterization of the associated constitutive interactions. Our objective in this and the following sections is to provide the reader with an idea of the diversity of the available models, their properties, and their applications in a qualitative and non-exhaustive manner; for much more detailed and technical presentations the interested reader is referred to the excellent reviews that have been recently presented in the literature (Noid, 2013; Kmiecik et al., 2016; Singh and Li, 2019; Nguyen et al., 2021).

Depending on the nature of the ingredients employed in the construction of the CG potential, particle-based models are usually divided in three main classes: knowledge-based, top-down, and bottom-up models (Noid, 2013).^7^

In the knowledge-based approach, the parameters of the CG potential are identified through statistical analyses performed over one or more experimentally resolved, static protein structures. To some extent, knowledge-based methods thus directly translate bioinformatic or “frequentist” information about the occurrence of specific local properties—such as side-chain affinities (Tanaka and Scheraga, 1976; Miyazawa and Jernigan, 1996; Bahar and Jernigan, 1997; Davtyan et al., 2012), backbone torsional angles (Betancourt, 2008; Kim et al., 2013), or hydrogen bond capabilities (O’Meara et al., 2015)—to the forces acting among the system’s CG effective sites. Top-down models, on the other hand, typically hinge on simple functional forms for the CG potential, the *a priori* choice of which is dictated by physicochemical intuition, and fine-tune their constituent parameters so as to reproduce a set of experimentally-measured meso-to macroscopic observables for the system at hand, including structural and/or thermodynamic ones (Monticelli et al., 2008; Coluzza, 2011; Najafi and Potestio, 2015; Dignon et al., 2018; Perego and Potestio, 2019; Dignon et al., 2019).

The two aforementioned CG strategies do not explicitly rely on the existence of a more fundamental model of the protein, in our case an all-atom force field; the problem of distilling the interactions among CG sites directly from those governing the microscopic constituents, so that the former become emergent properties of the latter, is addressed in bottom-up methodologies.

Bottom-up CG’ing stems from a rigorous statistical mechanics framework in which the high-resolution detail of a system is explicitly integrated out in favor of a lower-resolution representation (Rudzinski and Noid, 2011). This process results in an effective interaction among the CG sites, the potential of mean force (PMF), which in principle provides a complete, *faultless* description of the system as observed through the “CG glasses”, see Figure 3. The price one pays for the simplification is that the PMF is intrinsically many-body in nature: even if the energetic landscape of the original microscopic system comprises only pair potentials among its constituent atoms, once the resolution reduction is performed a whole hierarchy of interactions appears that involve, in addition to pairwise terms, triplets of CG sites, quadruplets, and so on (Dijkstra et al., 1999). These many-body components can play a key role in generating, and comprehend the origin of, the correct large-scale behavior of a system (D’Adamo et al., 2015; Menichetti et al., 2017), such as, in the case of proteins, secondary structure motifs (Kolinski et al., 1993; Derreumaux, 1999; Bereau and Deserno, 2009; Liwo, 2013; Sieradzan et al., 2017); at the same time, however, their presence makes the exact determination of a PMF largely unfeasible in practice, except for very simple microscopic models (Diggins et al., 2018).

The ultimate goal of bottom-up strategies has thus become the construction of increasingly accurate approximations to the correct result, achieved by relying on a wide variety of different theoretical techniques. Approaches exist that allow the explicit calculation of the set of interactions composing the low-resolution potential, including the aforementioned many-body terms, through a systematic decomposition of the PMF in terms of Kubo cluster cumulants (Liwo et al., 2014; Sieradzan et al., 2017; Liwo et al., 2020). Other methods focus instead on reproducing a subset of the system’s structural observables (Lyubartsev and Laaksonen, 1995; Soper, 1996; Tschöp et al., 1998; Mullinax and Noid, 2009a; Lyubartsev et al., 2010; Rudzinski and Noid, 2015) or aim at approximating the MB-PMF by means of variational approaches, either directly (Shell, 2008; Shell, 2016), or matching the many-body mean forces—that is, the gradient of the MB-PMF (Izvekov and Voth, 2005; Noid et al., 2008a; Noid et al., 2008b). In the case of structure-based techniques, the implicit assumption is that, if the model generates a set of important properties whose values are quantitatively in line with those of the high-resolution model, this is a sign of CG interactions reasonably approximating the MB-PMF; conversely, if the CG potential optimally reproduces the MB-PMF as in variational approaches, one can expect it to give rise to observables that match the AA ones.

Recently, many of these strategies have benefited from the introduction of machine learning protocols that aim at easing the construction and usage of bottom-up CG force fields; notable examples are, in the case of force-based methods, DeepCG for liquid water (Zhang et al., 2018) and CGnets for small peptides (Wang et al., 2019). Despite having been so far applied to relatively small systems, the promising results obtained suggest that machine learning techniques will soon grow to become a cornerstone in the parameterization of accurate CG potentials for biologically relevant macromolecules.

Irrespective of the parameterization workflow, CG interactions pose nontrivial conceptual challenges. In principle, the selection of a filter with a specific level of resolution allows one to *observe* all phenomena in the system that occur at a length scale equal to or larger than the characteristic size of the elemental CG units; in the construction of a CG model, though, it is the choice of the interactions that limits its ability to *reproduce* such phenomena. If the CG potential accurately reproduces the MB-PMF, all thermodynamical properties and observables of the system can be obtained, even if they originate from processes that take place at a scale below the resolution level of the model (Wagner et al., 2016; Lebold and Noid, 2019a; Lebold and Noid, 2019b; Dannenhoffer-Lafage et al., 2019). However, in practical applications it is not possible to calculate *all* many-body contributions that appear in the PMF, let alone embodying them into computationally manageable functional forms. In the construction of the CG potential one is thus doomed to rely on a limited basis set of interaction terms, commonly consisting in few-body ones, which leaves out high-order contributions; consequently, some effects of the removed DoF’s will not appear. *With a limited expansion of the MB-PMF, we thus expect that a reduction in the resolution level will correspond to a decrease in the spectrum of properties and phenomena that the model is able to predict.*

In some occasions it is reasonable to suppose that a particularly well-chosen representation of the system might lead to a substantial simplification of the interactions, e.g., by making many-body terms small or even negligible (D’Adamo et al., 2015): if this were the case, the MB-PMF could be expressed through simple interactions among few constituents, thus making the model simple to parametrize and understand. Alternatively, if the many-body nature of the PMF cannot be reduced or neglected, more complex interactions have to be incorporated, as it is done in the case of density-dependent potentials (Allen and Rutledge, 2008; Sanyal and Shell, 2016; Wagner et al., 2017; Sanyal and Shell, 2018; Rosenberger et al., 2019; DeLyser and Noid, 2019; Shahidi et al., 2020).

It is thus of paramount importance, for a successful usage of coarse-graining methods, to identify which is the simplest model—in terms of representation and interactions—that is capable of accurately reproducing the properties of interest. This problem will be addressed in Sections 5 and 6 of this work, where we will present an overview of examples taken from the literature. Specifically, in Section 5 we will focus on the interplay between resolution level and range of observable phenomena: we will discuss how, in general, by decreasing the former we limit the latter, but at the same time we gain access to larger length and time scales. The discussion will be restricted to the case of homogeneous CG representations, that is, models in which roughly the same level of resolution is employed throughout the whole protein structure.

The homogeneity constraint will be subsequently relaxed in Section 6, where we will focus on examples of hybrid models in which different levels of resolution are concurrently coupled in the description of the protein and/or the surrounding solvent. This approach is particularly suited for the study of phenomena localized on a well-defined region of the molecular structure: in such a case, the high-resolution level is typically dictated by the characteristic length scale of the phenomenon of interest, while the rest of the system can be described with a coarser, and consequently computationally less expensive, degree of detail.

## 5 Coarse-Grained Modeling: Resolution Level

In Section 4 we discussed how a reduction in the resolution level of a CG model can in turn lead to a limitation in the amount of emergent properties or phenomena the model is able to reproduce. Critically, this is not a mere consequence of the increase in the model’s smallest length scale; it also stems from our incapacity to parametrize CG potentials capable of comprehensively capturing the increasing amount of microscopic detail that gets integrated out.

In this section we will explicitly investigate this tradeoff by relying on a subset of protein CG models extracted from the literature (Saunders and Voth, 2012; Kmiecik et al., 2016; Singh and Li, 2019; Nguyen et al., 2021). We will restrict our analysis to homogeneous models and present them in order of decreasing resolution, moving from the most detailed CG representations all the way down to continuum pictures. For each resolution step, we will mention what phenomena the corresponding model is appropriate to investigate.

Before starting the discussion, however, a couple of remarks are in order. Firstly, we stress that the following list is not exhaustive. The CG models selected in this work serve the only purpose of providing the reader with a representative landscape of the possible descriptions employed to investigate biomolecular systems across a wide range of time and length scales.

Secondly, although a decrease in the resolution of the CG model should in principle correspond to an increase in the characteristic sizes of the analyzed systems and phenomena it can produce—thanks to the reduction in the associated computational cost—in the presented applications this will not always be the case. This is a consequence of our choice of ranking CG models according to their resolution level rather than their chronological appearance: more detailed and thus resource-intensive CG models, when recent, benefited from the modern explosion experienced in accessible computational power, enabling their applications to system sizes that only a few years earlier would have been unconceivable to address at such a high level of detail. The inverse correlation between resolution and accessible time/length scales should thus be interpreted as a trend rather than a rule.

**FIGURE 4:**
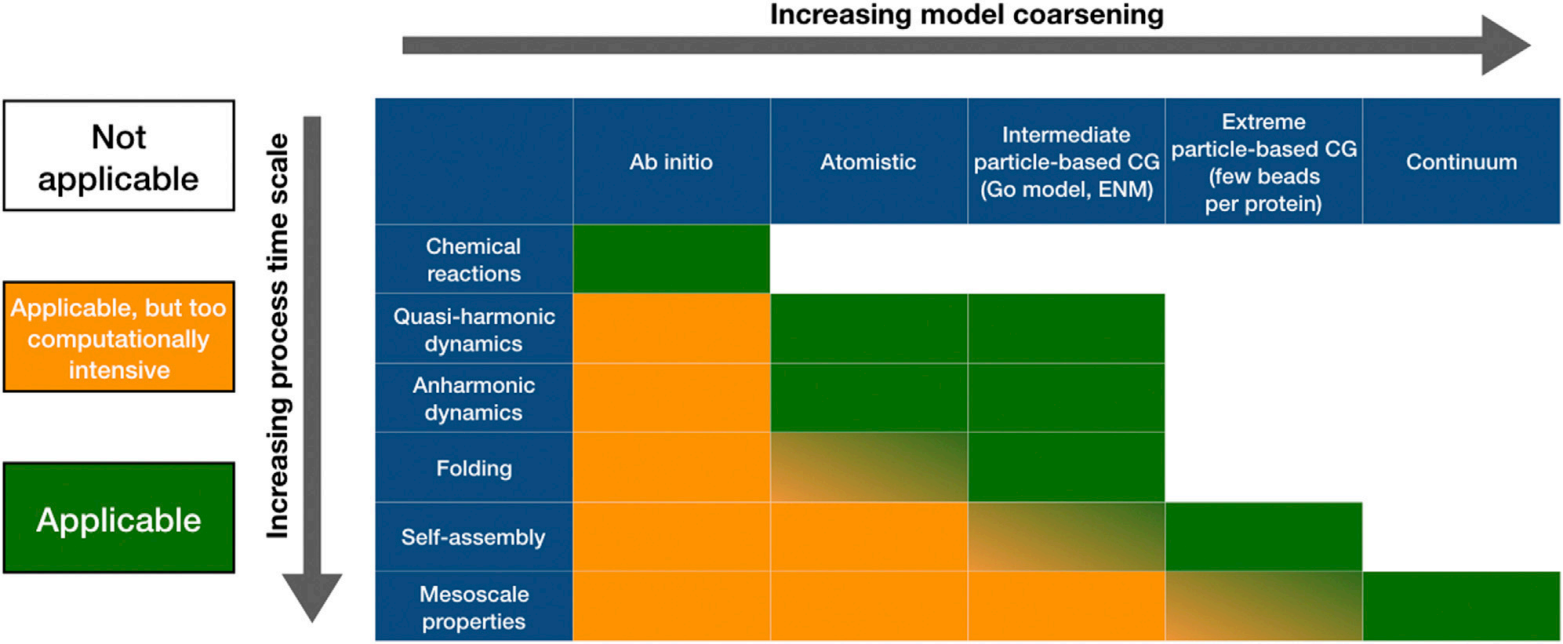
A schematic illustration of the relation between a model’s accuracy and its capacity of reproducing long time-scale phenomena. In principle, an extremely accurate model *might* reproduce all phenomena that take place at a characteristic length and time scale that lies above that of its fundamental constituents; however, practical limitations make its usage impossible beyond a certain limit. The coarser the model, the longer the time scale that can be achieved, at the expenses of a shorter and shorter list of processes that it can manage to produce.

To construct our hierarchy of CG models, in the following we will resort to a division in four main categories that account for similarities in the underlying CG’ing philosophies. Specifically, in Section 5.1 we will discuss explicit solvent CG models, whose elemental units aim at preserving, in a reasonable although approximate manner, the chemical features of the original microscopic components of the protein as well as those of the solvent in which the protein is immersed. Subsequently, in Section 5.2 this relatively high degree of local detail will be significantly reduced through the introduction of implicit solvent CG models. It is in this context that a decrease in resolution, combined with further simplifications in the associated interactions, more severely implies a bottleneck in the landscape of phenomena a specific model can capture. The residue-based decomposition of a protein that is common to both explicit and implicit solvent CG models will be then loosened in Section 5.3, where we will discuss Ultra-CG ones. Here, a single effective site becomes representative of group of residues, a small protein, up to an entire molecular complex. Finally, in Section 5.4 the particle-based CG’ing scheme will be abandoned in favor of protein models in the continuum. A schematic representation of this resolution-based hierarchy of CG force fields, providing information about which emergent phenomena each class of models can provide insights on, is presented in Figure 4.

### 5.1 Explicit Solvent Coarse-Grained Models

The uppermost rung of a hierarchy of CG models arranged in order of decreasing resolution is occupied by particle-based ones that account for the solvent environment in a simplified *but explicit* manner. Immersed in this CG solvent, an ensemble of beads is then employed to describe a protein, each bead being meant to encapsulate a small chemical moiety comprising few constituent atoms, thus resorting to a rather moderate level of CG’ing. Notable examples in this class of models include the popular SIRAH (Darrè et al., 2015; Machado et al., 2019) and MARTINI (Marrink et al., 2007; Monticelli et al., 2008) force fields, in which, within a relatively “granular” solvent, a quite conspicuous number of effective interaction sites is employed to represent a single amino acid composing the protein structure. Particular attention is further paid to approximately capturing the “local” chemical features and flexibility of amino acid side chains, so that several beads can be employed in their description. Overall, this fairly high level of detail can limit the computational speedup generated by these models, especially due to the presence of the solvent; at the same time, it often allows an almost one-to-one reconstruction, or backmapping, of the microscopic structure starting from the CG one (Darrè et al., 2015).

Interactions among the CG sites are parametrized to account for the average properties of the atoms they enclose, and include bonded as well as non-bonded contributions; in both SIRAH and MARTINI, the former are tailored so as to reproduce (a subset of) structural features, such as bond distances and the bending and dihedral angles between consecutive units. Different philosophies lie instead at the core of the determination of the non-bonded potentials: while SIRAH aims at providing an accurate description of the system electrostatics and sterics (Darrè et al., 2015; Machado et al., 2019), MARTINI mainly targets experimental free energies of partitioning of small chemical fragments between a polar and an apolar phase (Marrink et al., 2007; Monticelli et al., 2008). In both cases, the result of this overall parameterization protocol is a “dictionary” of CG building blocks, one per amino acid, that can be combined together to model the protein structure of interest and investigate its behavior.

The resolution level and chemical specificity characterizing the fundamental units of SIRAH and MARTINI enables their application to the investigation of large-scale conformational and/or thermodynamic properties of a system, as well as to problems in which the local detail, down to a sub-residue level, can play a crucial role on the system emergent phenomena: among these we mention the rearrangement of side chains; hydrogen bonding; and protein-solvent, protein-protein, or protein-substrate interactions. Despite the similar length scales characterizing the elemental units composing the two models, however, already at this limited degree of CG’ing the delicate interplay between resolution level and effective interactions has a considerable impact on the spectrum of observable phenomena. Restricting ourselves to one significant example, SIRAH was shown to be able to preserve the stability of proteins comprising α-helix as well as β-sheet elements in absence of explicit topological biases (Darrè et al., 2015). On the contrary, MARTINI requires secondary structure motifs to be enforced *a priori*, thus preventing its application in studies involving folding or general conformational rearrangements (Monticelli et al., 2008; Poma et al., 2017; Souza et al., 2020). While this limitation is commonly associated to the relatively low resolution at which the protein backbone is treated in MARTINI (one bead per peptide), it should rather be considered a direct consequence of the particular choice in the parametrization of the interactions: in fact, effective models exist that rely on MARTINI-like CG representations and are capable of stabilizing secondary structure elements without introducing ad hoc constraints (Alemani et al., 2010; Spiga et al., 2013).

This crucial difference naturally introduces a distinction in the class of phenomena on which the two models can provide insight. Specifically, SIRAH has been largely applied in the study of problems where structural properties, in combination with the local chemical detail, are pivotal: these include the dynamic behavior of disordered proteins (Ramis et al., 2019), the impact of post-translational modifications on protein structural stability (Garay et al., 2019), and the prediction of protein-protein binding free energies (Patel and Ytreberg, 2018). On the other hand, the parametrization of MARTINI is based on polar/apolar phase partitioning, which makes it particularly suited in the analysis of protein-membrane systems. Applications include the insertion and assembly of membrane proteins and protein-protein complexes in lipid bilayers (Bond and Sansom, 2006; Periole et al., 2012), the investigation of the effect of protein crowding on transmembrane diffusion (Javanainen et al., 2013), and the simulation of proteins in realistic membrane environments (Corradi et al., 2018). Recently, the model was also shown capable of predicting protein-ligand binding affinities with no prior knowledge of binding pockets or pathways (Souza et al., 2020).

### 5.2 Implicit Solvent Coarse-Grained Models

Explicit solvent CG models are required when, although by relying on a blurred microscope, an attempt of tackling all of the intricacies of a system’s local chemical maze is conducted. On the contrary, their level of resolution can be considered excessive when dealing with phenomena that take place at larger length scales, such as protein folding, conformational rearrangements, or self-assembly. Consider for example the case in which a net attraction/repulsion between pairs of amino acids constitutes the driving force of the macroscopic process; for this to emerge from the CG model, a much lower resolution than that of SIRAH or MARTINI might be sufficient, e.g., removing the solvent and describing each amino acid as an effective interaction unit.

In principle, such a procedure should come at the price of introducing a more complex (free-)energetic landscape among the elemental sites to compensate for the additional reduction in detail. This, however, is largely unfeasible in practice, and one typically relies on further approximations, such as the derivation of the low-resolution potential through the truncation of formal statistical mechanics series expansions (Liwo et al., 2014), or its *a priori* definition in terms of extremely simplified functional forms (Derreumaux, 1999; Voegler Smith and Hall, 2001; Bereau and Deserno, 2009; Cheon et al., 2010).

This discussion brings us to the second class of CG models within our hierarchical ladder, that is, *implicit solvent* ones. Here, as the name suggests, the solvent degrees of freedom are integrated out from the description, and one only accounts for the effect they *on average* exert on the proteins under investigation. Such proteins, on the other hand, are still decomposed in terms of the their constituent residues, albeit in an increasingly simpler form as the structural coarsening progresses. It is in this context that the correlation between resolution level, CG interactions, and range of observable phenomena becomes particularly strong: a decrease in the first is usually not balanced by an increase in the second, which in turn can result in a reduction of the third.

Among implicit solvent CG models, the more detailed ones aim at preserving the “chemical identity” of each amino acid. Since such information is inherently contained in the side chain, this directly translates into the usage of one or more explicit CG beads representing it and accounting for its chemical features, in addition to the effective sites that are employed to describe the peptide backbone. In analogy with the case of the explicit solvent models discussed in Section 5.1, the desired outcome is again a protocol in which the fundamental units embodying each amino acid type can be joined together to assemble the specific system under investigation. Examples of such *intermediate resolution* CG force fields are OPEP (Derreumaux, 1999; Maupetit et al., 2007; Sterpone et al., 2013), the one by Bereau and Deserno (BD) (Bereau and Deserno, 2009), PRIME (Voegler Smith and Hall, 2001; Cheon et al., 2010), AWSEM (Davtyan et al., 2012; Wu et al., 2018), and UNRES (Liwo et al., 2014; Sieradzan et al., 2017; Liwo et al., 2020).

The first model, OPEP, is characterized by a high degree of structural detail (Derreumaux, 1999; Maupetit et al., 2007; Sterpone et al., 2013). All the heavy atoms composing the protein backbone as well as the amide hydrogens are retained as CG sites, while a single bead describes the side chain of each amino acid—except for proline, which is represented by all its heavy atoms. Interactions among these fundamental units are then parametrized via a combination of structural, thermodynamic and knowledge-based approaches, and comprise conventional bonded and nonbonded contributions—e.g., harmonic or Lennard-Jones potentials—as well as terms that account for hydrogen bond capabilities and ion pair interactions. Interestingly, while the original version of the model neglected the solvent degrees of freedom, hydrodynamic interactions were later incorporated in OPEP by coupling it with a Lattice Boltzmann representation of the solvent (Sterpone et al., 2015). As for BD and PRIME, they lean on a similar CG mapping prescription to describe each amino acid, namely three beads for the backbone and one for the associated side chain. Notable differences exist, however, in the derivation of their constitutive interactions. In particular, in analogy with OPEP, BD is again defined in terms of a conventional basis set for the bonded and non-bonded interactions, whose fundamental parameters are tuned by combining structural and knowledge-based protocols (Bereau and Deserno, 2009). In addition to terms accounting for connectivity, steric repulsion, side chain affinities, and hydrogen bond capabilities, the BD force field aims at favoring the correct α/β secondary structure ratio through the presence of additional bonded potentials mimicking dipolar-like interactions that tend to stabilize β-sheet components. Furthermore, BD was later generalized to protein-lipid systems (Bereau et al., 2014). PRIME, on the other hand, resorts to a very crude interaction network in which extremely simplified potentials such as hard-sphere and square-well functions describe steric repulsion and bonding/attractive interactions among the effective sites, respectively (Voegler Smith and Hall, 2001). This choice enables the usage of discontinuous molecular dynamics (Rapaport, 1978; Bellemans et al., 1980), further speeding up simulations. Originally blind to the side chain chemical detail, PRIME was later generalized via a knowledge-based approach so as to capture their specificity (Cheon et al., 2010). In AWSEM, three CG sites, respectively located on the peptide C_*α*_, C_*β*_, and oxygen atoms, are employed to represent a single protein amino acid (Davtyan et al., 2012; Chen et al., 2016; Wu et al., 2018). Bonded potentials among the AWSEM CG units are then complemented with a complex network of nonbonded interactions: these include hydrogen-bonding terms, bioinformatic terms biasing the formation of local structures, ^8^ nonlocal terms describing contacts—either direct or water/protein-mediated—among distal residues along the sequence, and burial terms that aim at accommodating an amino acid into its preferential environment—e.g., the protein bulk or surface. The corresponding parameters are tuned via a combination of structural and knowledge-based approaches. AWSEM further enables the simulation of membrane proteins by relying on an implicit membrane potential (Kim et al., 2014). Finally, UNRES maps each amino acid onto three CG sites, namely the C_*α*_ atom, the center of the peptide bond, and the side chain, the latter being described as an ellipsoid of revolution (Liwo et al., 2014). Only the last two elements, however, are explicit effective interaction sites, while the C_*α*_ sites only serve the purpose of tracing the protein geometry. Interactions among the UNRES building blocks are then parametrized through a rigorous bottom-up procedure: the potential of mean force of the system is expanded in a truncated series of Kubo-cluster cumulants, which enable the derivation of the multi-body interactions acting among the CG sites in a systematic manner (Liwo et al., 2014; Sieradzan et al., 2017; Liwo et al., 2020).

The computational speedup generated by OPEP, BD, PRIME, AWSEM and UNRES enables their application to problems whose characteristic time and length scales were, until recently, prohibitively large to be effortlessly accessed by conventional all-atom simulations. Specifically, OPEP was largely employed in the context of folding and structure prediction of isolated proteins, protein-ligand and protein-protein complexes (Wei et al., 2004; Shen et al., 2014; Lamiable et al., 2016; Kynast et al., 2016), in aggregation studies (Lu et al., 2012; Nasica-Labouze and Mousseau, 2012), as well as to investigate the structure of long, intrinsically disordered amyloid monomers (Nguyen and Derreumaux, 2020). Moreover, the introduction in OPEP of a Lattice Boltzmann solvent paved the way for its exploitation to analyze hydrodynamic effects on biomolecular systems, including the behavior of proteins under shear flow (Sterpone et al., 2018) or the impact of molecular crowding on the dynamics of protein suspensions (Sterpone et al., 2014). Applications of the BD model encompass the investigation of folding processes, including the analysis of the interplay between secondary and tertiary structures in cooperative folding (Bereau et al., 2010), as well as peptide aggregation phenomena (Bereau and Deserno, 2009). Given the additional computational gain provided by its discontinuous potentials, PRIME was instead extensively exploited to investigate the behavior of large-scale systems, especially in the context of aggregation of fibrils in presence or absence of fibrillation seeds or inhibitors (Nguyen and Hall, 2004; Cheon et al., 2011; Wang et al., 2017; Wang and Hall, 2018). In addition to protein folding (Jin et al., 2020), applications of AWSEM include the investigation of protein-protein association (Zheng et al., 2012) and fibrillar aggregation processes (Zheng et al., 2016; Chen et al., 2020), as well as the analysis of the static and dynamic behavior of intrinsically disordered proteins (Wu et al., 2018; Lin et al., 2019). The incorporation of an implicit membrane potential in AWSEM enabled it to provide insight on the folding behavior of transmembrane proteins (Lu et al., 2018) and protein assemblies (Truong et al., 2015). Finally, while UNRES was originally applied to perform protein structure prediction via energy minimization (Liwo et al., 1999), subsequent MD-based studies include the investigation of folding processes (Liwo et al., 2005), self-assembly of protein complexes (Sieradzan et al., 2012), fibrillar aggregation (Rojas et al., 2010; Rojas et al., 2017), as well as conformational transitions in molecular chaperones (Gołaś et al., 2012).

The power of the intermediate resolution CG models lies in their transferability, that is, the possibility of employing them to provide insight on the behavior of systems that were not directly involved in the models’ parameterization. It follows that particular care must be taken as far as meso-to macroscopic properties are concerned; while these can be explicitly included in the construction of the effective potential, for the latter to be transferable the introduced restraints should be flexible enough so as not to bias the model predictions toward very specific outcomes, associated to particular systems. This requirement is especially evident in the case knowledge-based approaches, in which abstraction of the interaction parameters from the stable conformation of a specific protein—conformation that is here interpreted as the emergent property participating in the parameterization of the CG potential—is achieved by performing a statistical analysis over an ensemble of structures (Tanaka and Scheraga, 1976; Miyazawa and Jernigan, 1996; Bahar and Jernigan, 1997; Betancourt, 2008; Kim et al., 2013; O’Meara et al., 2015). It is thus possible, and indeed often advantageous, to design transferable implicit solvent CG models tackling well-defined large-scale problems; at the same time, one should make their constitutive ingredients as general as possible, so as to enable the characteristic phenomenon of the system of interest to arise from the model, without the need of imposing it a priori. On the other hand, one might need implicit solvent CG models that are more severely bound to a subset of known macroscopic properties associated to a specific biomolecule. In this case, the model could be asked, e.g., to reproduce the experimentally resolved tertiary structure of a particular system. The emergent property now directly represents an input of the CG’ing protocol.

One could clearly resort to standard CG’ing strategies and develop a dedicated effective model in which these conditions are satisfied (Izvekov and Voth, 2005; Rudzinski and Noid, 2011; Shell, 2016); this often lengthy parameterization procedure, however, should at least in principle be repeated from the ground up every time a new system is investigated, for which the same kind of external piece of information is available. It is therefore highly desirable to construct CG models that rely on more “intuitive” interaction potentials and are easily generalizable to arbitrary systems through a minimal fine-tuning. The particular choice of the phenomenological potential will play a pivotal role in defining the class of phenomena the model can *additionally* provide insight on. The simplification of the interaction network typically goes on par with an additional reduction in the resolution level and chemical detail, with every amino acid composing the molecule being now described as a single interaction site.

A notable example of this second class of implicit solvent CG models is represented by *structure-based* ones, such as Gō-like models (GLM) (Hills and Brooks, 2009; Takada, 2019) or elastic network models (ENM) (Sanejouand, 2013; Togashi and Flechsig, 2018). Here, the external macroscopic input involved in the construction of the effective CG potential is the static, either stable or metastable, three-dimensional spatial conformation assumed by the protein of interest. Both GLM and ENM describe the interaction among the elemental CG units in terms of very general functional forms, tailored to *reproduce* the target structure but easily applicable to arbitrary ones; the complexity and richness of the basis set, however, significantly decreases while moving from GLMs to ENMs, generating a crucial impact on the spectra of phenomena these two classes of models can respectively capture.

Gō models originally represented a protein as a self-avoiding walk on a lattice (Taketomi et al., 1975). Large-scale structural information enters GLMs through attractive interactions occurring between pairs of sites that, although distant along the protein sequence, are in direct contact in the native conformation. Despite this extremely crude description, such models are capable of driving a protein to spontaneously fold toward its native state (Go, 1983). The lattice formulation was later extended to the continuum enabling the use of MD simulations (Clementi et al., 2000). Here, a protein is represented by sites located on its C_*α*_ atoms and interacting via simple potentials whose functional form is borrowed from standard all-atom force fields. The folded conformation is enforced in both bonded and non-bonded interaction terms: the former are parametrized by setting the equilibrium structural parameters equal to the distances and bond/dihedral angles of the protein native state; non-bonded contributions are instead conceptually akin to the lattice version of the model, so that general, unspecific attractive (resp. repulsive) interactions occur between residues that form (resp. do not form) a native contact. In both cases the strength of the interaction parameters is *independent of the residues’ chemical detail*; this condition, together with the C_*α*_ mapping prescription, was later relaxed in subsequent generalizations, which relied on more chemically-realistic functional forms for the interactions (Karanicolas and Brooks, 2002), as well as quasi-atomistic descriptions of the biomolecule (Whitford et al., 2009).

Due to their extreme simplicity and flexibility, GLMs have a long and successful history in the field of protein folding (Hills and Brooks, 2009; Hu et al., 2017; Takada, 2019). Furthermore, the original native-centric standpoint was later extended to account for the presence of multiple (meta)stable conformational basins, allowing transitions between them at greatly reduced computational cost (Best et al., 2005; Okazaki et al., 2006). Applications in this context range from the investigation of conformational rearrangements of “simple” proteins (Lu and Wang, 2008), all the way up to, e.g., large-scale molecular motors (Hyeon et al., 2006; Kanada et al., 2013).

Sticking to a structure-based CG’ing protocol but further reducing the complexity of the interaction basis set one encounters elastic network models (Sanejouand, 2013; Togashi and Flechsig, 2018). ENMs stem from the pioneering observation, made by Monique Tirion (Tirion, 1996), that the low-frequency dynamics of globular proteins, *in the vicinity of their native conformation*, can be accurately reproduced by replacing the system’s complex interaction network by a set of Hookean springs of *equal strength* connecting neighboring atoms up to a given cutoff distance. CG equivalents of this original version of the model have been subsequently developed, which typically retain one or two atoms per amino acid (Atilgan et al., 2001; Micheletti et al., 2004). Structural information is more strictly enforced in ENMs compared to GLMs, preventing the study of processes such as folding or tertiary structure rearrangements. As for the latter, however, it was shown that ENMs are able to capture at least the essential, early-stage behavior of a protein’s conformational changes, further cementing their role as a fundamental building block in the edifice of mesoscopic CG modeling of biomolecules (Tama and Sanejouand, 2001; Petrone and Pande, 2006). Moving away from protein simulations, the simplicity of ENMs allowed their application to the investigation of the low-energy fluctuations of complex systems where all-atom as well intermediate resolution CG models would prove computationally too demanding, ranging from macromolecular motors such as ribosomes (Tama et al., 2003) up to entire viral capsids (Tama and Brooks, 2005; Grime et al., 2016).

### 5.3 Ultra Coarse-Grained Models

The class of models presented in Sections 5.1 and 5.2, although characterized by a gradual decrease in the level of detail, always rely on a residue-based decomposition of a protein, in which only one or few effective interaction centroids describe *each* amino acid composing the biomolecule. To push the applicability of particle-based CG models to the investigation of phenomena occurring at even larger time and length scales, one possibility is that of resorting to ultra coarse-graining (UCG) methods. Here, each CG site becomes representative of larger chemical entities, be that few residues, whole proteins or even entire molecular complexes (Chu and Voth, 2006; Sept and MacKintosh, 2010; Zhang et al., 2017). Several examples of UCG models, ranging from more “chemically accurate” to more heuristic ones, have been presented in the literature. While more traditional applications typically focus on single proteins (Zhang et al., 2017; Zhang et al., 2020), UCG methods have provided impressive insights into the behavior of overwhelmingly complicated macromolecular structures (Saunders and Voth, 2012; Hagan and Zandi, 2016), including actin filaments (Chu and Voth, 2006), bacterial flagella (Arkhipov et al., 2006a), and viral capsids (Arkhipov et al., 2006b; Nguyen et al., 2009; Grime et al., 2016).

As pointed out in Dama et al. (2013), from a conceptual point of view UCG models pose notable additional challenges compared to their more detailed counterparts, which are, as it is the case for the previously discussed studies, often overlooked in the construction of the UCG effective interaction potential of a system. Specifically, as the structural coarsening progresses, several internal states of the system can end up being mapped onto the same CG configuration. For instance, let us consider the case of a macromolecular complex, a whole protein of which is represented as a single UCG site. If the protein undergoes a conformational rearrangement between two states that leave the CG site coordinates unaltered, both states contribute to the energetic landscape of a single CG macrostate and, as far as the model is concerned, they are indistinguishable. At the same time, the rearrangement could play a key role in the generation of the macroscopic phenomenon of interest, and it would thus be desirable to construct a UCG model able to discriminate the two conformational basins. To tackle the problem of constructing CG models for systems possessing internal states, Voth and coworkers have recently developed an extremely elegant Theory of Ultra Coarse-Graining (UCGT) in a series of works (Dama et al., 2013; Davtyan et al., 2014; Dama et al., 2017), to which we refer the interested reader. While applications of this theory have been, to our knowledge, so far limited to relatively high-resolution CG representations of liquids, UCGT represents an extremely promising framework for the development of accurate UCG models of biologically relevant macromolecules.

### 5.4 Continuous Models

Particle-based CG models share the fundamental common feature of tracking the dynamics of a system through each of its mesoscopic constituent degrees of freedom, or effective interaction sites. As an extreme act of coarse-graining, such a scheme can be completely abandoned in favor of representations that treat the whole macromolecular body as a continuous medium subject to the laws of hydrodynamics.

In this perspective, starting from the observation that a protein in its folded, globular conformation behaves as a viscoelastic solid (Wang and Zocchi, 2011), Harris *et al.* introduced the Fluctuating Finite Elements Analysis (FFEA) scheme for macromolecular simulations (Oliver et al., 2013). In FFEA, fluctuations of a biomolecule around its native conformation are described through hydrodynamic observables, with the evolution of the system in response to stress being obtained by means of finite element analysis. Notably, in addition to elastic and viscous factors, the effect of thermal noise is directly incorporated into the protocol by means of an appropriate stress tensor. The absence of an atomistic level of detail clearly sets a dramatically large lower bound to the length scales achievable by FFEA; on the other hand, this method represents a promising opportunity for pushing the analysis of biological systems to truly meso-to macroscopic scales. Originally applied to the prediction of the dynamics of globular proteins close to their native states (Oliver et al., 2013), FFEA was later employed to analyze the behavior of complex macromolecular systems such as molecular chaperones (Solernou et al., 2018), conformational transition of molecular motors (Richardson et al., 2014; Hanson et al., 2015; Richardson et al., 2020; Hanson et al., 2021), and the effect of the application of stretching and torsional forces on the structural stability of antibodies (van der Heijden et al., 2020).

A straightforward application of FFEA to the case of highly elongated biomolecules such as thin, rod-like structures is difficult because of the variety of length scales characterizing the conformational variability of these systems. To tackle the problem, Welch *et al.* recently introduced the Kirchhoff biological rod algorithm (KOBRA), a fluctuating rod model designed to perform continuum simulation of slender molecules (Welch et al., 2020). In KOBRA, a thin system is represented as an elastic material curve subject to thermal noise, whose dynamic equations of motion are solved based on a discretization in terms of straight rods connecting a set of nodes. The first application of the model on elongated protein complexes showed promising results; furthermore, the coupling of KOBRA with FFEA suggests the possibility of generating mesoscopic, continuous models of biomolecular systems comprising globular as well as slender components.

## 6 Coarse-Grained Modeling: Resolution Distribution

The first historical applications of hybrid multiscale models of biomolecules trace back to the 1970s, with the works of Warshel and Karplus (1972) and, a few years later, of Warshel and Levitt (1976). These works, coupling a quantum mechanical and a classical description, led the foundation for the quantum mechanics/molecular mechanics (QM/MM) methodologies (Amaro and Mulholland, 2018; Magalhães et al., 2020), whose relevance was recognized by the attribution of the Nobel prize in Chemistry to Karplus, Warshel, and Levitt in 2013. The development of QM/MM approaches opened the way for the coupling of lower resolution levels for the investigation of phenomena happening at increasingly larger length scales. In this section, we present examples of such coupling schemes and their range of applications. These include processes of ligand binding studied with hybrid atomistic/coarse-grained resolutions, or protein conformational changes reproduced by the integration of CG scales at different levels of detail. In addition, examples of a dual description of the solvent (atomistic/CG or atomistic/continuum) are reported: they allow the construction of a larger simulation box, representing a computationally efficient solution for finite-size effects. Importantly, all these cases require the definition of the resolution domains during the phase of simulation set-up, on the basis of some previous knowledge of the system. This issue is overcome by the use of coarse-graining as an informative tool, as explained in Section 7.

### 6.1 Coupling Quantum Mechanical–Classical Atomistic Models

In QM/MM, a computationally expensive quantum mechanical approach is used to simulate only a subset of atoms, where a classical force field may fail: a typical example is the active site of an enzyme, where a chemical reaction takes place. The other components of the system, including the rest of the protein and the solvent, are treated in a less computation-intensive way by molecular mechanics. The classical, atomistic description of the largest part of the system allows the simulation of full proteins in their natural environment, either the solvent or the lipid bilayer; however, the time-consuming quantum mechanical calculations—even though restricted to a small number of residues—limit the time scale spanned, which typically covers a few hundreds of picoseconds.

Despite this limitation, which can nonetheless be alleviated by the application of enhanced sampling techniques (Yang et al., 2019), the QM/MM method is having an increasing impact on the study of biomolecules (Lonsdale et al., 2013; Lonsdale et al., 2014; Tyzack et al., 2016), mostly enzyme-ligand complexes. For instance, QM/MM simulations proved useful to compute binding free-energy profiles and barriers for enzyme-catalyzed reactions (Barnes et al., 2013), and to characterize binding kinetics (Haldar et al., 2018). Moreover, QM/MM plays a key role in drug design for the discovery of covalent inhibitors (Lodola et al., 2008; Ranaghan et al., 2014), small organic molecules that steadily inactivate the target protein by forming a covalent bond.

Although enzymatic reactions have been the primary target of QM/MM studies, the approach proved to be effective also for the investigation of proton transfer events, where the excess positive charge is propagated through a network of hydrogen bonds dynamically connecting water molecules, protein residues, and/or cofactors. Since this process involves breaking and forming covalent bonds and charge delocalization, a QM description is required at least in the region where the transfer takes place. Recent applications include the study of ion channels, such as the Cl^−^H^+^antiporter ClC-ec1 (Chiariello et al., 2020). Here, DFT-based QM/MM simulations and well-tempered metadynamics (Barducci et al., 2008) free energy calculations were performed, contributing to explain the transport inhibition in ClC anion/proton exchangers. Another less obvious field of application of QM/MM simulations is the computational study of metallodrugs—namely coordination and organometallic complexes, typically containing platinum, silver, gold, vanadium, or iron ions (Palermo et al., 2016). The variety of coordination modes, bond breaking and formation, ligand exchange reactions, charge-transfer, and polarization effects in these molecules requires a QM description of the drug and its binding site on the biomolecular target. A widely studied case is the mechanism of action of cysplatin, one of the most effective and broadly used chemotherapeutic agents (Calandrini et al., 2015).

A recent methodological progress in QM/MM simulations is the development of a Hamiltonian adaptive multiscale scheme (Boereboom et al., 2016). As solvent molecules diffuse in and out of the reactive region, they are gradually included into (and excluded from) the QM computation. This was later implemented along with state-of-the-art path integral simulation techniques, which allow for the calculation of quantum statistical properties, and ring-polymer and centroid molecular dynamics, which allow the calculation of approximate quantum dynamical properties (Kreis et al., 2017). In another definition of QM/MM adaptive scheme, the boundaries of the QM region change during the simulation, adapting themselves to the reaction site (Mones et al., 2015). These advancements pave the way for further methodological developments in the QM/MM field.

### 6.2 Coupling Atomistic–Coarse-Grained Models and Beyond

Biological phenomena do not always involve the breaking/formation of chemical bonds, which require an accurate description of the electronic structure. It is the case, for instance, of non-covalent protein-protein and ligand-protein interactions, including the vast majority of drug discovery applications, where the designed drug is not supposed to undergo any chemical reaction once accommodated in the protein binding site. If the domains involved in the interaction are known in advance, e.g., from experimental evidence or previous computational analysis, one can additionally exploit the inherently multiscale nature of the problem to build a hybrid atomistic/coarse-grained (AA/CG) set-up, where the atomistic detail is retained only in the region of interest (in the above example, the binding site of a receptor). The rest of the macromolecule is instead treated at a lower, coarse-grained resolution, bringing the immediate advantage of a reduced computational cost.

This general idea gave rise to a variety of approaches, where the details of each method (namely, the resolution distribution and the parameterization of interactions) are specifically designed to tackle the system under investigation. Examples range from the multi-resolution model of a polyamide melt (Gowers and Carbone, 2015), where only the amide groups involved in the formation of the hydrogen bonds are maintained at atomistic resolution, to multimeric complexes including both proteins and nucleic acids, as in Villa et al. (2004), Villa et al. (2005), and Dans et al. (2010). In the latter case, a multi-resolution simulation of the lac repressor protein from *E. coli* and a 107-bp-long DNA segment is performed, where the protein and the two bound operators are described atomistically, while the DNA loop is modeled as an elastic ribbon connecting the terminal base pairs of the DNA operators.

In most of AA/CG applications the size of the atomistic region is larger than a single chemical moiety, but substantially smaller than the protein itself. This is the case of ligand-binding multiscale studies, where an atomistic resolution is required for only a few protein residues. In the work by Fogarty and coworkers (Fogarty et al., 2016), an ENM representation of the hen egg-white lysozyme is coupled with an atomistic description of the active site, with and without the inhibitor di-N-acetylchitotriose. The same model has been employed by Fiorentini and coworkers (Fiorentini et al., 2020) with the aim of assessing the accuracy of a hybrid AA/CG description of the protein for binding free energy calculations.

A hybrid method specifically designed for the study of ligand-protein interactions is the so-called Molecular Mechanics/Coarse-Grained approach (MM/CG) (Neri et al., 2005; Neri et al., 2006; Leguèbe et al., 2012; Tarenzi et al., 2019). In its first version (Neri et al., 2005), MM/CG is validated on cytoplasmic enzymes, whose catalytic site is represented atomistically, while the rest of the protein is described at a CG resolution according to a Gō-like model. Despite the absence of explicit solvent, the method showed a good agreement between the RMSF of the MM/CG simulations and the fully atomistic ones, and a good overlap between the subspaces of the most relevant eigenvectors computed with MM/CG and atomistic MD.

The MM/CG method was then applied to membrane receptors of pharmacological relevance. In particular, with the introduction of a surface potential surrounding the transmembrane region of the protein and mimicking the interaction with the lipid bilayer (Leguèbe et al., 2012), the MM/CG was specifically tailored for predicting binding poses in low-resolution models of membrane proteins, such as homology models of G-protein-coupled receptors (GPCRs). The paucity of experimental structural information and the low sequence identity between members of the family lead to models with inaccurate side chain orientations, which may introduce biases in fully atomistic simulations: in such cases, coarse-graining part of the receptor allows atomistic residues in the binding site to relax more easily to the biologically functional conformation. Atomistic water molecules in the extracellular side, hydrating the binding site, are confined by a repulsive potential. This approach has been widely tested on bitter taste receptor GPCRs (Schneider et al., 2018; Fierro et al., 2019), and recently implemented in a webserver pipeline (Schneider et al., 2020).

In the latest implementation of the method (Open Boundary MM/CG) (Tarenzi et al., 2019), the multi-resolution model of the protein is coupled to an adaptive resolution description of the solvent through the Hamiltonian adaptive resolution (H-AdResS) scheme (Potestio et al., 2013) (see Section 6.3 for further details). Water is modeled with atomistic accuracy in the two hemispheres capping the intracellular and extracellular parts of the receptor, and free diffusion is ensured with a surrounding reservoir of CG water molecules. The improved hydration model leads to the simulation of a rigorous statistical ensemble and enables accurate binding free energy calculations for a drug design purpose (Korshunova and Carloni, 2021).

We conclude this subsection mentioning multi-resolution models where the two or more resolutions concurrently employed are coarse-grained, that is, lower than atomistic. These approaches aim at reproducing the large-scale conformational dynamics of large biomolecules in a particularly efficient manner, and are especially easy to rationalize. Proteins have been modeled as networks of a small number of CG sites, fewer than the total number of residues (Doruker et al., 2002; Kurkcuoglu et al., 2004; Eom et al., 2007), and unevenly distributed along the primary structure. The partitioning among resolution levels can be performed on the basis of previous knowledge of the working of the system functions: this is the case of the multiscale network model (Jang et al., 2009): here, the fine-grained region is constituted by specific functional sites represented at the residue level as an ENM; the remaining regions are described at a lower resolution, including only a subset of the C_*α*_ atoms as interaction sites.

In other approaches, the choice of the level of resolution and its distribution along the protein structure is not so obvious. This is the case of the essential dynamics coarse-graining (ED-CG) (Zhang et al., 2008; Zhang et al., 2009), where residues undergoing collective dynamics are represented by pseudo-nodal points. Such CG sites are determined through a variational approach, with the objective of reproducing the protein’s essential dynamics.

This last example illustrates in a rather clear manner the relationship between the filter or mapping on one hand, and the resulting model on the other. The definition of the CG sites is not determined by their chemical structure or identity (as it is the case for residue-to-bead mappings), but rather it is a consequence of their *emergent properties*, such as the internal flexibility. This, in turn, implies a non-uniform assignment of atoms to beads, as different parts of the protein can show different degrees of a given property, so that each CG site represents an arbitrary number of atoms—or, alternatively, the resolution of the model varies non-uniformly along the structure in terms of mass, number of atoms, or chemical identity. More importantly, the mapping is not assigned by the modeler from the top down: it is identified by the system itself as the solution to a minimization process. This is to say, a cost function is defined whose argument is the mapping and whose minimum is the *optimal* mapping. The idea that the system “informs the modeler” about which representation of itself is the most appropriate (given certain criteria) represents a crucial step forward in the process of modeling, and bears important consequences on the interpretation of its outcomes. Section 7 of this paper is devoted to exploring these ideas.

### 6.3 Multiscale Schemes Tailored for Solvent Description

We conclude this section with an overview of those multi-resolution approaches that have been applied to liquids and diffusive systems, rather than to bonded structures whose parts have a fixed resolution.

Needless to say, the most important liquid in biology is water. Water molecules often play a direct role in biological processes, such as ligand binding and enzymatic catalysis, by establishing stable non-bonded interactions with protein residues and/or ligands. At the same time, bulk solvent undoubtedly represents the computationally most expensive component of the simulation box. Several multiscale schemes have been designed in order to tackle this duality; they are based on the common idea that water in the hydration shell of a biomolecule requires an atomistic description, while bulk water can be described at a coarser resolution. However, such schemes pose the problem of enabling proper diffusion of solvent molecules across regions at different resolution, while keeping the overall thermodynamic equilibrium under control. This issue is tackled, e.g., in Szklarczyk et al. (2015) through the so-called “flexible boundaries for multiresolution solvation” (FBMS). Here, the spatial partitioning between atomistic and coarse-grained solvent is enforced by means of half-harmonic distance restraints, which attract atomistic molecules to the surface of the solute and repel the CG beads. A restraint-free region at intermediate distances enables the formation of a buffer layer, where the atomistic and CG solvents can mix freely.

An alternative is given by those methods that allow solvent molecules to smoothly change their resolution on the fly when transitioning between an atomistic region and a CG region; these include the adaptive resolution scheme (AdResS) (Praprotnik et al., 2005) and the Hamiltonian adaptive resolution scheme (H-AdResS) (Potestio et al., 2013). In both cases, solvent molecules are free to diffuse between regions at different resolution, without constraints; in so doing, they pass through a hybrid resolution layer, where interactions between molecules are governed by an interpolation of atomistic and CG forces (in case of AdResS) or potentials (in the case of H-AdResS). The interpolation scheme is defined by a position-dependent transition function, which smoothly couples the two domains. Moreover, tailored correction forces can be automatically calculated and applied to the molecules in the hybrid region, in order to ensure a uniform density profile across the simulation box.

The relevance of such approaches in the context of biomolecular simulations has been assessed by studying ubiquitin at fully atomistic resolution in a multi-resolution AdResS solvent (Fogarty et al., 2015), and atomistic proteins atox1 and cyclophilin J in an H-AdResS solvent (Tarenzi et al., 2017). In both works, each CG water molecule is represented as a single bead located on the molecule’s center of mass. CG particles interact through a potential derived from Iterative Boltzmann Inversion (Reith et al., 2003; Rosenberger et al., 2016), which reproduces the centre-of-mass radial distribution function of the atomistic solvent; the protein is placed at the center of the atomistic region, which is shaped as a sphere. A study on the effect of the high-resolution region radius on the solute and the hydration solvent was also performed. In Kreis et al. (2016), a similar approach is employed, however, the shape of the high resolution region is self-adjusting during the course of the simulation, following the conformational changes of an atomistic polypeptide during folding. The adaptive resolution representation of the solvent served also for the calculation of solvation free energies of side-chain analogues, using the AdResS (Fiorentini et al., 2017) or H-AdResS (Korshunova and Carloni, 2021) scheme. Further applications of adaptive resolution simulation methods include the coupling of atomistic water with a supramolecular, MARTINI-style model (Zavadlav et al., 2019), or even an ideal gas representation (Kreis et al., 2015), which can also provide an innovative solution for solvation free energy calculation, by pulling the solute from the atomistic solvent region to the CG one (Heidari et al., 2019).

A natural evolution of particle-based multiscale approaches toward even coarser resolutions is the coupling of atomistic solvent and continuum representations, which aims at extending the range of applicability of such models to system sizes beyond those reachable by particle-based models alone. Several examples exist of simulation schemes including atomistic and continuum descriptions for the simulations of water (Brünger et al., 1984; Beglov and Roux, 1994; Im et al., 2001; Lee et al., 2004; Deng and Roux, 2008; Wagoner and Pande, 2011; Wagoner and Pande, 2013; Petsev et al., 2015). Attempts of triple-scale simulation of liquid water have also been performed, by concurrently coupling atomistic, CG, and continuum models (Delgado-Buscalioni et al., 2009; Zavadlav et al., 2018). Applications of an atomistic/continuum representation of the solvent for biomolecular studies have been performed in Wagoner and Pande (2018), where the boundary between the explicit/continuum solvent models can adapt itself in response to the conformational fluctuations of the atomistic peptide simulated; and in Hu et al. (2019), for a multi-resolution simulation of protein diffusion in water under a steady shear flow.

## 7 On Choosing the Optimal Resolution Level and Distribution, and on Modeling as an Analysis Tool

In the previous sections we showed how coarse-graining techniques model soft matter systems, proteins in particular, using a plethora of simplified representations, each one characterized by its level of detail. In addition, several methods have been developed to concurrently employ, in the same simulation setup, models at different resolution, so as to provide a small subregion with an accurate description and the remainder of the system with a computationally efficient one. In both cases, the level of detail and its distribution is usually determined *a priori* on the basis of various characteristics (chemical identity, biological function, intuition), depending on the usage one does of the model. Recently, however, interest has grown around the idea of allowing the system itself to decide the “best” coarse-grained description of it. Clearly, the notion of “best” is relative, and it necessarily has to answer to the question *best for what?*

In this final section we report on the recent attempts to find the optimal resolution of a biomolecule, namely the “most appropriate” number and selection of degrees of freedom to describe it, together with their spatial distribution. These two concepts are deeply intertwined and several studies suggest the existence of a link among the optimal resolution, the distribution of detail assigned in the coarse-grained model, and the relevant properties of the system of interest. This connection has its roots in the philosophy behind bottom-up CG modeling, which assumes that the properties of a system should emerge from the behavior of a statistical mechanics-based, simplified model obtained through the (exact) integration of a subset of its degrees of freedom. Usually, this concept of “behavior” refers to the time evolution of the CG system and its conformational space sampling, which enable one to comprehend and understand it. Here, we argue that the process of simplification (mapping) itself can provide hints to non-trivial features of the high-resolution model. This hypothesis has immediate consequences, such as the conversion of coarse-graining methods into analysis tools, a change of paradigm that could constitute a valuable instrument for the analysis of high-resolution, fully atomistic representations of biomolecules.

In bottom-up CG modeling, the choice of the CG mapping has proved to be critical for the properties of interest to emerge systematically (Mullinax and Noid, 2009b; Rudzinski and Noid, 2011). This idea is pushed forward by Rudzinski and Noid (Rudzinski and Noid, 2014), who quantitatively rationalize how the quality of the modeling is influenced by the quality of the mapping. Specifically, the authors group the configurations sampled in a MD simulation into *n* (*m*) distinct molecular states of the high-resolution (low-resolution) system; as the low-resolution macrostates clearly depend on the choice of the mapping scheme, Rudzinski and Noid posit that the most informative CG representation should generate a bijective correspondence between atomistic and CG molecular states. This approach allows, in principle, to estimate the optimal level of resolution as well as its distribution. It is thus the system itself that informs the modeler about its low-resolution description that maximizes the consistency with the high-resolution behavior.

This promising paradigm is at the heart of a recent work by Fiorentini and coworkers (Fiorentini et al., 2020), in which a protein-ligand system is considered and the relationship between the binding free energy and the chosen level of resolution is quantified. The authors consider several hybrid atomistic–coarse-grained representations of the protein by treating a variable number of amino acids at the all-atom level. The resulting values of binding free energy are compared with the atomistic reference, showing that the accuracy of the dual-resolution model does not necessarily increase with the spatial extension of the atomistic region. This result suggests the existence of a system-specific, optimal number of amino acids that should be modeled with high detail in such hybrid schemes.

In general, then, the idea has started to emerge that a macromolecular system admits one or more *optimal* reduced models, that is, simplified representations in terms of which it (viz. its high-resolution model) can be *observed* with a marginal loss of information in spite of a loss of detail. Furthermore, it appears more and more evident that such an optimal representation cannot, in general, be uniform: the degree of fidelity with which the original, high-resolution structure is reproduced in the simplified model can vary from point to point, in parallel with the system’s chemical, mechanical, dynamical, and functional properties.

Foley and coworkers (Foley et al., 2015; Foley et al., 2020) have pioneered the analysis of the CG model spectrum in a formal and systematic way. In Foley et al. (2015) they considered a one-bead-per-residue Gaussian network model (GNM) of proteins as the reference, high-resolution representation; then, taking advantage of the exact integrability of GNMs, they performed a systematic *decimation* of the system’s beads to investigate how reduced models at varying degrees of resolution manage to reproduce fluctuations and correlations of the original model. In so doing, they showed that the information loss that is inherent in the process of coarse-graining is not a monotonic function of the resolution, as an optimal value of the latter was found for which the information content per CG bead (quantified by an appropriate measure) exhibits a maximum. These works thus highlighted the relation between the informativeness of a representation and its resolution *level*.

The impact of resolution *distribution* was later studied by Koehl and coworkers, also in this case making use of ENMs: the *Decimate* (Koehl et al., 2017) algorithm progressively reduces the resolution of a biomolecule by creating a hierarchy of increasingly simplified models, in the spirit of the renormalization group theory. As expected, such CG mappings show an uneven distribution of detail: in the case of globular proteins, for example, optimal models tend to concentrate atoms on the surface of the molecule, thus heavily coarse-graining the inner region—whose mechanical properties require fewer degrees of freedom to be aptly reproduced. A related approach is employed in a work by Diggins et al. (2018): here, the authors identify the CG beads that produce a coarse-grained ENM whose Hamiltonian interaction matrix is as close as possible, measured according to an appropriate distance, to the high-resolution, atomistic ENM. The proposed selection of atoms proves to outperform a random assignment in terms of several observables, such as the intra-block dynamics fraction.

Most of the mentioned approaches can be grouped under the umbrella of methods to optimize the SLRs of a biomolecule in order to improve the capability of the reduced models to faithfully reproduce the atomistic properties of interest. We now summarize the existing methods that, acting as pure filters, focus only on the choice of the SLR without considering the parametrization of the effective interactions.

The first prominent attempts at finding the most informative reduced description of a biomolecule can be ascribed to Voth and coworkers, who employed the $\chi^{2}$ residual of essential dynamics to estimate the optimal number and partitioning of coarse-grained sites for large protein complexes (ED-CG) (Zhang et al., 2009; Zhang and Voth, 2010; Sinitskiy et al., 2012). In particular, in Sinitskiy et al. (2012) this $\chi^{2}$ is subject to a constrained minimization, in which the addition of a CG site to a simplified description of a molecule is accepted only if there is a substantial gain in information about the system. Related works (Li et al., 2016a; Li et al., 2016b; Wu et al., 2020) by Xia and colleagues start from the ED-CG method to develop several protocols for the determination of the optimal representations of biomolecules. In Li et al. (2016a) the authors introduce the stepwise optimization with boundary constraint (SOBC) algorithm to enhance the numerical performances of ED-CG (Zhang et al., 2009; Zhang and Voth, 2010) on large proteins. Subsequently (Li et al., 2016b) they propose to maximize the ENM pairwise fluctuations between atoms that are mapped to different CG sites (fluctuation maximisation). The resulting reduced models, once equipped with simple, harmonic interactions, are capable of matching the large-scale fluctuations of the corresponding fine-grained counterparts. More recently, Wu et al. (2020) adopt a combination of ED-CG and internal clustering validation indices to estimate the proper number of sites to coarse-grain proteins. Their results suggest that the appropriate number of C_*α*_ atoms to be preserved in a simplified model should lie between one half and one fourth of the total.

Multiple examples of the application of CG’ing methods to analyze simulation data of biomolecules rely on quasi-rigid domain decomposition (Hinsen, 1998; Aleksiev et al., 2009; Potestio et al., 2009). Polles et al. (2013) employed a quasi-rigid domain decomposition of several viral capsids to single out their fundamental mechanical blocks; once validated on a dataset of known viruses, this method is used to formulate predictions about structures whose mechanical subunits had not been characterized yet. Following a similar approach Morra et al. (2012) studied MD trajectories of three representatives of the heat shock protein 90 (Hsp90) family, simulated with and without substrates. They observed that, when the protein is partitioned in as few as three quasi-rigid domains, the relative rigid-like movements of the latter can account for a significant fraction of the system fluctuations, thus allowing to pinpoint two *optimal axes* for rigid rotations of the domains. In turn, the position of these hinges was shown to correspond to two interfaces: while the biological importance of one of them had already been assessed, the other one was hitherto unknown, thus highlighting a potentially druggable functional site.

These remarkable results prove that it is possible to exploit CG methodologies to perform a detailed analysis of the fundamental aspects of an atomistic system. Nevertheless, it is important to notice how these approaches rely on the examination of *mechanical* properties of the system of interest; although they certainly represent simple, intuitive variables to look at, such features do not seem to be as fundamental as the underlying problem they are applied to. Examples of more profound approaches exist that aim at optimizing the SLR of biomolecules in a systematic way (Delvenne et al., 2010; Chen and Habeck, 2017; Boninsegna et al., 2018; Wang and Gómez-Bombarelli, 2019). Delvenne et al. (2010) rank SLRs according to the quality of the corresponding partitioning induced on the protein graph. Chen and Habeck (2017) propose a Bayesian procedure that extracts the optimal SLR from a single macromolecule or cryo-EM map. Boninsegna et al. (2018) combine time-averaged diffusion maps (Banisch and Koltai, 2017) and Markov State Models (Bowman et al., 2013) to select groups of atoms that are mutually close (coherent) over a conformational basin. Wang and Gómez-Bombarelli (2019) employ a variational autoencoder to learn a set of latent CG variables (that is, a SLR) from the atomistic configuration (FLR): in the decoding process the SLR aims at reconstructing the original FLR in a deterministic procedure.

**FIGURE 5:**
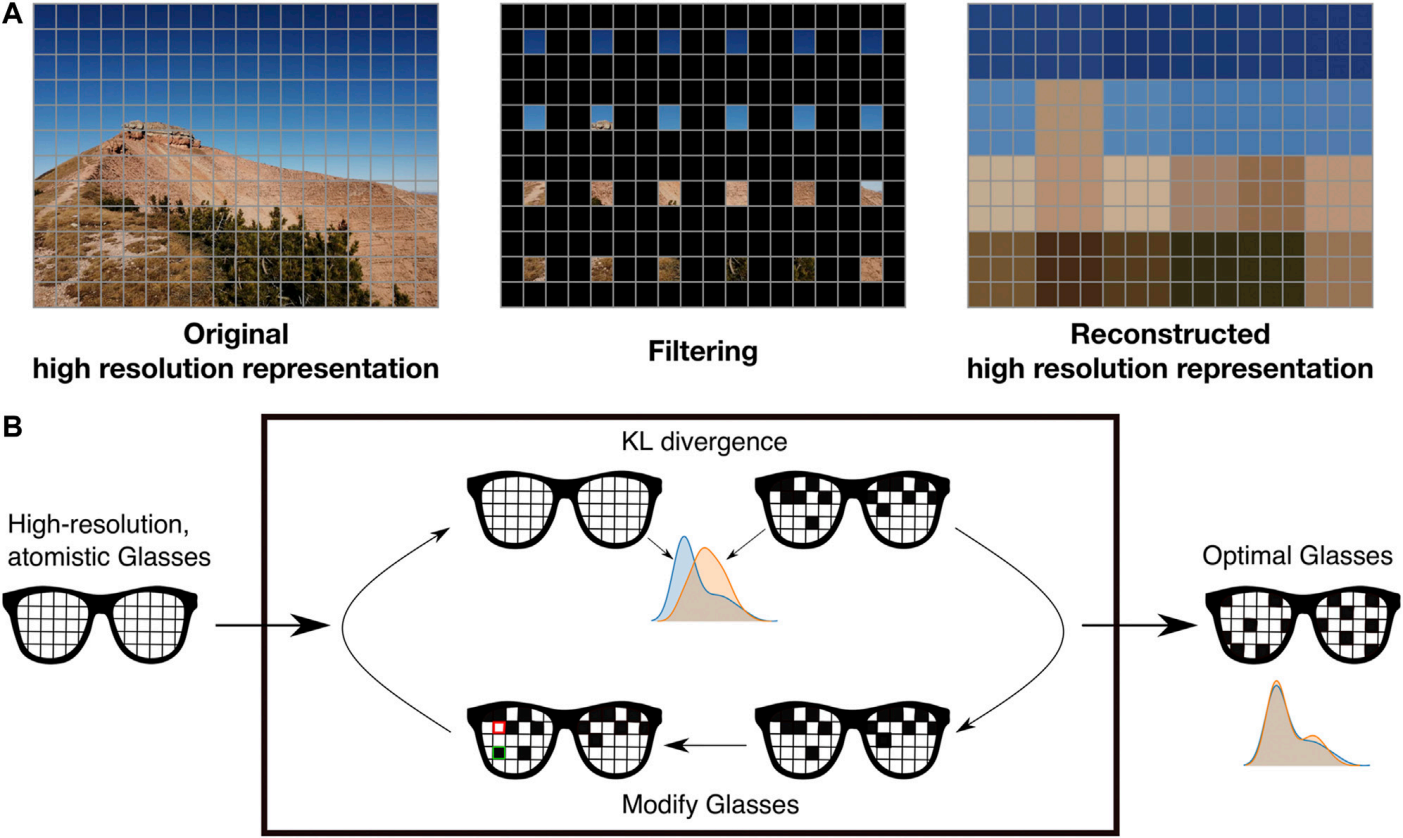
**(A)** The mapping entropy is defined as the Kullback-Leibler distance between the atomistic, fully detailed probability distribution ***(left panel)*** and its blurred reconstruction ***(right panel)***, obtained from a reduced description obtained neglecting a subset of degrees of freedom ***(middle panel)***. **(B)** The algorithmic implementation of the method introduced by Giulini and coworkers (Giulini et al., 2020) iteratively refines the CG mapping until convergence: the resulting, optimal SLR generates a reconstructed FLR that adheres as much as possible to the original one.

In the spirit of searching for a more significant and informative metric to link the FLR with the space of associated SLRs, some of us proposed a method (Giulini et al., 2020; Errica et al., 2021) that aims at optimizing the choice of the CG mapping through the minimization of the information loss between the description given by the all-atom model and its reduced representation. More specifically, the approach relies on the calculation of the *mapping entropy* $S_{map}$ (Shell, 2008; Rudzinski and Noid, 2011; Foley et al., 2015), which is the “distance”, in a Kullback-Leibler sense, between the all-atom Boltzmann distribution and its projection onto the CG space. Figure 5A illustrates a comparison of these distributions.

Given a reference all-atom MD simulation and a CG mapping, this protocol optimizes the latter until a (necessarily local) minimum of $S_{map}$ is reached (see Figure 5B). CG mappings obtained from independent minimisations of $S_{map}$ share features that are connected to the relevant biological properties of proteins. Moreover, the resolution is not uniformly assigned across the structures, but rather it is distributed to preserve the maximum amount of information about the original, atomistic description.

Since the calculation of $S_{map}$ can be computationally time-consuming, some of us (Errica et al., 2021) have proposed a machine learning model to accelerate the assessment of the quality of a coarse-grained mapping. This improvement allows one to estimate the correct density of states of the system (expressed in terms of the mapping entropy) by means of the Wang Landau sampling scheme, a calculation that would be computationally intractable without such machine learning-based acceleration.

In conclusion, all the works showcased here reflect the emergence of a profound need in the computational biophysics community: that of a strategy to build a faithful simplified representation of a molecular system in an entirely unsupervised manner. In standard coarse-graining recipes, such reduced descriptions must be equipped with proper effective interactions in order to *generate data*. However, the impressive development of techniques to enhance the performances of atomistic simulations is making this necessity less and less pressing. In contrast, the huge amount of high-resolution data produced at each MD run these days might benefit from the capacity of CG models to serve as powerful instruments to *make sense of the data*.

## 8 Discussion

In this review we have presented a broad, though certainly incomplete, overview of the ideas and motivations behind coarse-grained modeling. The construction of a model of a physical system, simple enough to be employed and understood while detailed enough to enable nontrivial insight, is one of the core activities of science in general. In the study of soft and biological matter, this need becomes particularly pressing and complex, as the advantages of generality, symmetry, and universality one enjoys in areas such as particle physics or statistical mechanics of critical systems lose ground in favor of specificity, peculiarity, and non-transferability; these latter characteristics, however, are those that confer to soft matter its spectacular spectrum of properties.

In such a varied and diverse scenario, one needs a comparably large toolbox of models and analysis techniques to crack the code of the relation among the constituents of a system, their arrangement and relations, and the emergent properties. The term “coarse-grained models” encompasses indeed such a variety, providing descriptions of the same system at different levels of resolution and detail, and serving as instruments to *produce* a given behavior as well as techniques to *analyze* it.

During the past few decades the vast majority of the effort has been put in the usage of coarse-grained models *in lieu* of more detailed, but also computationally more expensive descriptions; the recent impressive advancements of computer science are releasing pressure from this need, and all-atom simulations can now be performed of systems whose size and time scales were yesterday achievable by low-resolution models only.

However, the feasibility of large-scale all-atom simulations is not really putting coarse-grained models out of their job, but rather it is making them change employment: indeed, the extraordinary amount of data generated by such simulations is, in general, all but trivial to understand, and appropriate methods of analysis are required to make this information intelligible. The knowledge acquired in the development of effective low-resolution models thus proves especially useful in discriminating the signal from the noise.

To conclude, based on the presented analysis of the development of protein modeling throughout the decades, we foresee that a bright future lies ahead of coarse-graining: there will always be an inpatient necessity of simple models to investigate complex phenomena, as the curiosity of researchers is bound to lie beyond the capacity of their tools; complementarily, as more and more systems will be viable for accurate and detailed simulations, the need will grow for algorithmic, unsupervised methods to climb the mountain of data, reach its top and say *we understand*.

## Footnotes

1
https://www.atomicheritage.org/history/human-computers-los-alamos.

2 The SM is incomplete as it does not incorporate the general-relativistic theory of gravity; furthermore, it is an effective theory as it holds for energies lower than the Planck scale (see e.g., Burgess (2020)).

3 As it will be detailed in the following sections, filters and SLRs are referred to in the literature as *mappings* and *mapped representations*, respectively.

4 In a currently very popular language, one might call this a feature extraction process.

5 Monte Carlo (MC) is another popular simulation technique, which, however, found less space in the all-atom investigation of biomolecular systems; its main disadvantages, when compared to MD, are the inefficiency for exploring the configurational space of large biomolecules, the slowness of the convergence rate, and the lack of information about the time evolution of structural events (Adcock and McCammon, 2006).

6 Note that this definition does not straightforwardly apply to the case of adaptive resolution models, *vide infra*.

7 We stress that this sharp distinction is getting more and more smeared as the field of CG modeling steadily evolves: indeed, complementary ingredients extracted from each of the three aforementioned classes are often combined together in parametrizing the CG potential of a protein.

8 We exclude from the discussion the AAWSEM force field developed by Wolynes and coworkers (Chen et al., 2016; Chen et al., 2017) in which the local structure biasing terms are obtained through explicit all-atom MD simulations of fragments of the protein under investigation, thus rendering the model non transferable.
